# Targeted epigenetic silencing of UCHL1 expression suppresses collagen-1 production in human lung epithelial cells

**DOI:** 10.1080/15592294.2023.2175522

**Published:** 2023-02-22

**Authors:** Dan-Dan Wu, Andy T. Y. Lau, Yan-Ming Xu, Marjan Reinders-Luinge, Mihaly Koncz, Antal Kiss, Wim Timens, Marianne G. Rots, Machteld N. Hylkema

**Affiliations:** a Department of Pathology and Medical Biology, University of Groningen, University Medical Center Groningen, Groningen, The Netherlands; b GRIAC Research Institute, University of Groningen, University Medical Center Groningen, Groningen, The Netherlands; c Laboratory of Cancer Biology and Epigenetics, Department of Cell Biology and Genetics, Shantou University Medical College, Shantou, P. R. China; d Institute of Biochemistry, Biological Research Centre, Eötvös Loránd Research Network (ELKH), Szeged, Hungary; e Doctoral School of Biology, Faculty of Science and Informatics, University of Szeged, Szeged, Hungary

**Keywords:** UCHL1, smoking, DNA methylation, epigenetic editing, extracellular matrix

## Abstract

Ubiquitin carboxyl-terminal hydrolase L1 (UCHL1) is highly expressed in smokers, but little is known about the molecular mechanism of UCHL1 in airway epithelium and its possible role in affecting extracellular matrix (ECM) remodelling in the underlying submucosa. Since cigarette smoking is a major cause of lung diseases, we studied its effect on UCHL1 expression and DNA methylation patterns in human bronchial epithelial cells, obtained after laser capture micro-dissection (LCM) or isolated from residual tracheal/main stem bronchial tissue. Targeted regulation of UCHL1 expression *via* CRISPR/dCas9 based-epigenetic editing was used to explore the function of UCHL1 in lung epithelium. Our results show that cigarette smoke extract (CSE) stimulated the expression of UCHL1 *in vitro*. The methylation status of the UCHL1 gene was negatively associated with UCHL1 transcription in LCM-obtained airway epithelium at specific sites. Treatment with a UCHL1 inhibitor showed that the TGF-β1-induced upregulation of the ECM gene COL1A1 can be prevented by the inhibition of UCHL1 activity in cell lines. Furthermore, upon downregulation of UCHL1 by epigenetic editing using CRISPR/dCas-EZH2, mRNA expression of COL1A1 and fibronectin was reduced. In conclusion, we confirmed higher UCHL1 expression in current smokers compared to non- and ex-smokers, and induced downregulation of UCHL1 by epigenetic editing. The subsequent repression of genes encoding ECM proteins suggest a role for UCHL1 as a therapeutic target in fibrosis-related disease.

## Introduction

Ubiquitin carboxyl-terminal hydrolase L1 (UCHL1; also known as protein gene product 9.5, PGP9.5), is a member of ubiquitin C-terminal hydrolases (UCH) [1], and as such regulates ubiquitin modification and controls intracellular protein degradation. In control lung tissue, UCHL1 was first described to be expressed in airway neuroendocrine cells [2], which are rare, innervated airway epithelial cells accounting for <1% of the lung epithelium population. In addition to being present in neuroendocrine cells of both non-smokers and smokers, UCHL1 was highly expressed in ciliated epithelial cells of smokers [2,3]. In cancer, UCHL1 has been described to act as a tumour suppressor, as it was silenced in several cancer types, such as prostate cancer, ovarian cancer, and nasopharyngeal carcinoma [4,5]. However, high expression of UCHL1 was found in primary lung tumours (both small and non-small cell lung cancer) [6,7]. UCHL1 is also involved in cell apoptosis, neuronal differentiation, synaptic functions, and contextual memory [8,9]. Although higher epithelial expression of UCHL1 has been reported in smokers, the underlying molecular mechanism is not fully understood, and a role for UCHL1 in the pathogenesis of lung diseases remains to be determined.

Cigarette smoke, a leading cause of chronic lung diseases, is closely associated with airway remodelling and fibrosis [10] through the production of transforming growth factor-1 (TGF-β1) by airway epithelial cells [11,12]. Prompted by the proposed potential role for UCHL1 as a therapeutic target in fibrosis [13–16], we explored the function of UCHL1 in extracellular matrix (ECM) remodelling by human lung epithelial cells. In this study, we investigated the effect of smoking on the expression of UCHL1 and studied the DNA methylation pattern of the UCHL1 gene, particularly in airway epithelium obtained by laser capture micro-dissection (LCM). We explored the possible role of UCHL1 in smoking-related lung diseases *via* chemical inhibitor treatment and conventional plasmid-based upregulation in cell lines. Furthermore, CRISPR/dCas9-mediated epigenetic editing of UCHL1 (using a catalytically inactivated Cas9) [17] – was employed to up- and downregulate the expression of UCHL1 to further validate these findings.

## Results

### Upregulation of UCHL1 in smokers

**Figure 1.**
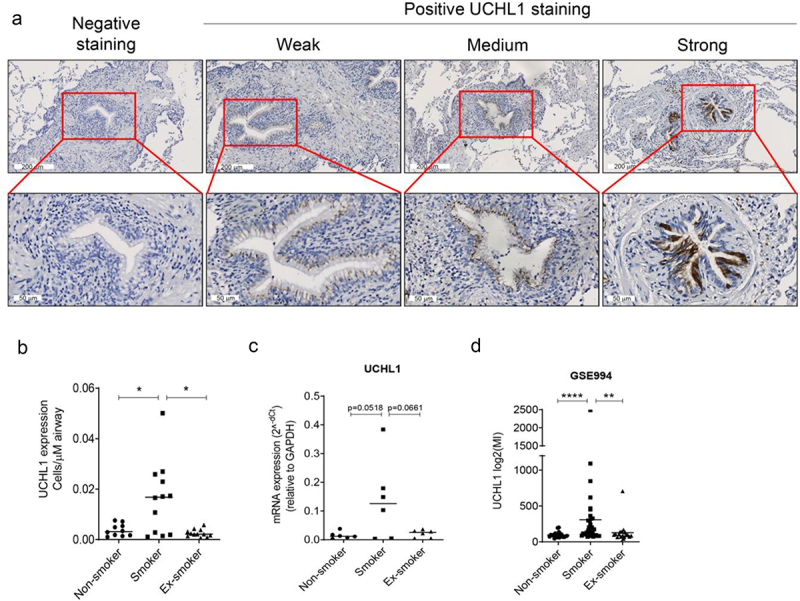
Smokers have more cells with strong-expressed UCHL1 than ex- and non-smokers. (a) Representative pictures of negative and positive UCHL1 staining in lung tissue. The positively stained UCHL1 cells in bronchial airways had either a weak, medium, or strong expression. (b) Quantification of manually counted cells with a strong expression of UCHL1 in airway epithelium of non-smokers (n = 10), smokers (n = 12), and ex-smokers (n = 12). Significance was analysed by nonparametric *Mann-Whitney U*-test. (c) mRNA expression of UCHL1 in total RNA isolated from non-smokers, smokers, and ex-smokers by LCM-collected epithelium (n = 6). Significance was analysed by one-way ANOVA. (d) UCHL1 mRNA expression was increased in current smokers from GEO dataset. UCHL1 expression was measured by microarray through brushing from intra-pulmonary airways (the right upper lobe carina) and scrapings from the buccal mucosa, which is from smoking and non-smoking volunteers (including 34 smokers, 23 non-smokers, and 18 ex-smokers). Gene expression is shown as log2 (MI). Significance was analysed by nonparametric *Mann-Whitney U*-test. *p < 0.05, **p < 0.01, ****p < 0.0001. Abbreviations: MI, Microarray Intensity.

To confirm previous studies, which reported higher UCHL1 expression in smokers, immunohistochemical staining was performed to detect UCHL1 expression in lung samples from non-smokers, current smokers, and ex-smokers. Staining of UCHL1 in airways showed a pattern of weak, medium, and strong expression in epithelial cells (where UCHL1 was expressed throughout the whole cell) (Figure 1a). Next, the cells with strong expression were counted to compare the effect of current smoking on the number of UCHL1 expressing cells, in comparison to non-smoking and ex-smoking. Samples derived from current smokers had significantly higher numbers of UCHL1 positive cells, compared with those of non-smokers and ex-smokers (4.4-fold, *p* = 0.043 and 6.4-fold, *p* = 0.017, respectively; Figure 1b). Furthermore, in a small pilot experiment, analysis of UCHL1 mRNA expression in LCM-dissected airway epithelium from current smokers showed higher UCHL1 expression compared with non-smokers or ex-smokers (*p* = 0.052 and *p* = 0.066, respectively, Figure 1c). Indeed, 4 out of 6 samples derived from smokers displayed higher UCHL1 expression than the highest expression observed in the control groups. This trend in mRNA expression differences was consistent with the protein expression data in Figure 1b. Moreover, mRNA expression in the LCM samples measured by real-time qRT-PCR correlated with protein expression assessed by immunohistochemistry staining (r = 0.5958, *p* < 0.01), as shown in Supplemental Figure 1. Interestingly, cell transcriptomic data from a GEO database (GSE994) [18] confirmed that expression of UCHL1 is higher in airway brushes of smokers compared to non-smokers or ex-smokers (Figure 1d).

### DNA methylation patterns in the promoter region of UCHL1

**Figure 2.**
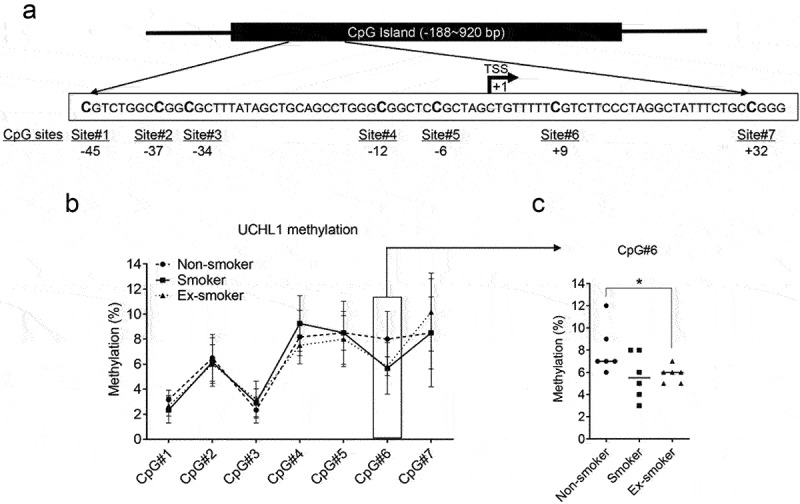
UCHL1 promoter methylation and mRNA expression in airway epithelium from non-smokers, smokers, and ex-smokers obtained by LCM. (a) Schematic representation of the CpG island of the UCHL1 gene, outlining the CpG sites around the TSS. The TSS is shown as +1. (b) Bronchial airway epithelium from non-smokers, smokers, and ex-smokers was collected by LCM, (n = 6 per group) and DNA methylation of seven CpG sites was analysed by pyrosequencing. Data represent the connected median methylation levels at different CpG sites (n = 6). (c) The percentage of methylation at CpG site #6 (+9 bp) for each group is indicated. Significance was analysed by nonparametric *Mann-Whitney U*-test, *p < 0.05.

To investigate the correlation between the DNA methylation status of the UCHL1 promoter and UCHL1 expression in samples of smokers *versus* non-smokers, first the methylation state of seven CpG sites around the transcription start site (TSS) was assessed in six lung cell lines (Figure 2a). The DNA methylation levels of individual CpG sites varied between 2% and 91% in these cell lines (Supplemental Figure 2A) and negatively correlated with UCHL1 mRNA expression levels (Supplemental Figure 2B and Supplemental Table 1). Next, the seven CpGs were interrogated for their methylation levels in the LCM samples. The average methylation levels of the CpGs obtained for 18 samples ranged from 2.7% to 9.1% between these groups with different smoking status (Figure 2b). Compared to non-smokers, the methylation level of CpG site #6 was lower in ex-smokers, but this was not observed in current smokers (Figure 2c). In these patient samples, the average methylation levels of these seven CpGs negatively correlated with mRNA expression (r = −0.7063, *p* = 0.0011, Supplemental Figure 2C). Methylation of two of the seven CpGs (CpG site #1 (45 bp upstream) and CpG site #7 (32 bp downstream of the TSS)), correlated negatively with UCHL1 mRNA expression levels when samples were combined from all three groups (r = −0.5964, *p* = 0.027 and r = −0.5559, *p* = 0.050, respectively, Table 1). Intriguingly, when analysed within each group, the methylation level of CpG#3 (34 bp upstream of the TSS) strongly correlated with mRNA expression levels of UCHL1 (r = −0.9258, *p* < 0.0001) (Table 1) in samples derived from ex-smokers.

**Table 1.** Correlation of UCHL1 mRNA expression and DNA methylation.

Correlation of mRNA expression with methylation		All	Non-smoker	Smoker	Ex-smoker	Non-smoker and ex-smoker
CpG#1 − 45 bp	r	**−0.5964**	−0.2777	−0.5768	−0.8760	−0.6109
	p-value	**0.027**	1.000	0.550	0.066	0.058
CpG#2 − 37 bp	r	−0.2777	−0.3714	−0.4928	−0.1234	−0.38
	p-value	0.794	1.000	0.900	1.000	0.575
CpG#3 − 34 bp	r	−0.1350	−0.3339	0.6179	**−0.9258**	−0.5771
	p-value	1.000	1.000	0.733	**<0.0001**	0.076
CpG#4 − 12 bp	r	−0.4427	−0.1739	−0.5161	−0.6761	−0.4670
	p-value	0.197	1.000	0.850	0.200	0.311
CpG#5 − 6 bp	r	−0.2586	−0.0294	−0.3339	−0.5768	−0.3441
	p-value	0.901	1.000	1.000	0.550	0.625
CpG#6 + 9 bp	r	−0.1715	0.4140	−0.2899	−0.1852	−0.07966
	p-value	1.000	1.000	1.000	1.000	1.000
CpG#7 + 32 bp	r	**−0.5559**	−0.6088	−0.7714	−0.5218	−0.1201
	p-value	**0.050**	0.600	0.308	0.900	1.000

### Cigarette smoke extract (CSE) stimulates the mRNA expression of UCHL1

**Figure 3.**
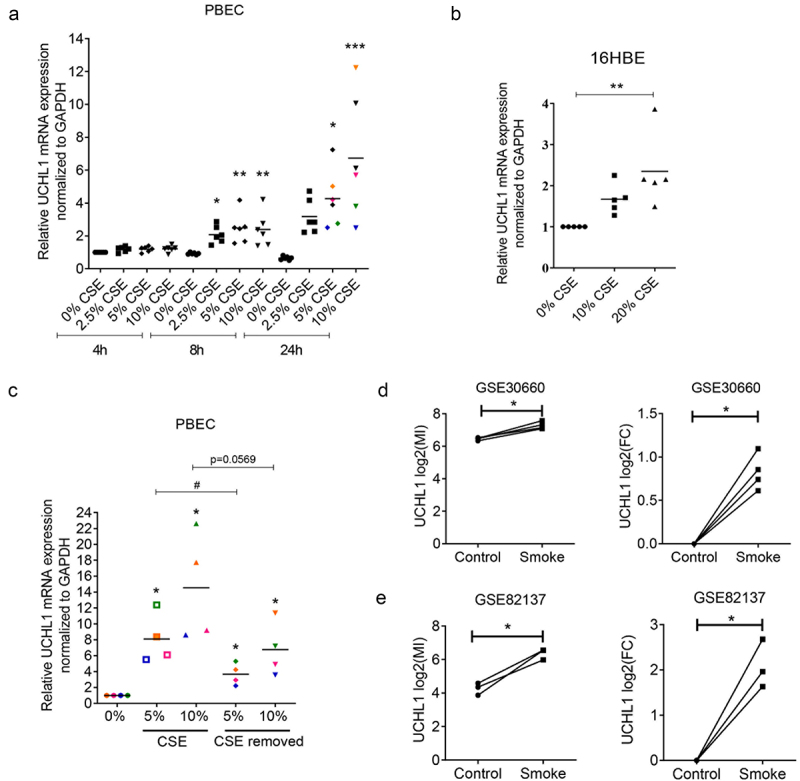
CSE stimulates the expression of UCHL1. (a) PBECs from lung transplant donors were seeded and treated without or with 2.5–10% CSE. Cells were harvested for RNA isolation at indicated time points (n = 6). Data from different doses of CSE were normalized to 0% at 4 h. Significances of 2.5%, 5%, and 10% CSE were analysed through comparing with 0% for each time point. (b) 16HBE cells were seeded and serum-deprived overnight, then treated without or with 10–20% CSE in EMEM without serum for 24 h (n = 5). Significance was analysed by one-way ANOVA (Kruskal-Wallis nonparametric test). (c) PBECs were sham-exposed or exposed with 5–10% CSE for 24 h. After 24 h incubation, CSE treated groups were exposed to indicated concentration of CSE for an additional 24 h, whereas CSE-removed groups were rinsed and replaced with complete growth KSFM medium for additional 24 h (n = 4, each colour represents a different donor, same as indicated in (A)). Cells were collected after 48 h incubation and subjected to RNA isolation. Significance (*) was analysed by paired *t*-test compared with untreated control. Significance (^#^) between CSE-treated group and CSE-removed group was analysed by paired *t*-test. (d, e) UCHL1 expression was altered upon smoke exposure in air–liquid interface co-culture model from GEO databases. UCHL1 expression was measured by microarray in bronchial epithelial cells grown at air-liquid interface (ALI) and exposed to gaseous cigarette smoke. (d) 30 minutes exposure to whole cigarette smoke or no challenge (n = 4) on four separate days and (e) 48 minutes of exposure on one day with whole cigarette smoke or air and then rested for 24 hours (n = 3). Gene expression is shown as log2 (MI) or log2 (FC) compared to control. Significance was analysed by two-tailed unpaired *t*-test. Abbreviations: FC, Fold Change. ^#^*p* < 0.05, *p < 0.05, **p < 0.01, ***p < 0.001.

To assess the direct effect of cigarette smoke on UCHL1 mRNA synthesis, primary bronchial epithelial cells (PBECs) from six donors as well as immortalized bronchial epithelial cell line (16HBE) were challenged with different doses of CSE. We observed a dose- and time-dependent effect of CSE on the upregulation of UCHL1 expression in both PBECs and the 16HBE cell line (Figure 3a and b) suggesting a role for UCHL1 to mediate the cellular response to CSE. However, short term (24 h) CSE exposure did not significantly modulate methylation of UCHL1 at CpG sites #1–6 (data not shown).

To further investigate whether the upregulation of UCHL1 depends on the actual presence of cigarette smoke, four samples were exposed to CSE for 24 h treatment after which cells were again exposed to CSE for 24 h or allowed to recover by incubating them in complete growth medium for another 24 h. Compared to untreated control cells, the expression of UCHL1 was increased significantly not only in cells upon continuous exposure to CSE for 48 h, but also in cells exposed to CSE for 24 h, followed by 24 h recovery (Figure 3c). Of note, the four samples that were exposed to both treatment regiments showed a similar fold-induction of UCHL1 expression in the two different sets of experiments (Figure 3a and c). Additionally, RNA-seq data from two independent publicly available GEO databases (GSE30660 and GSE82137) confirmed that the expression of UCHL1 is induced by gaseous cigarette smoke exposure in PBECs obtained from seven donors tested in the air–liquid interface (ALI) culture model with or without the removal of smoke (Figure 3d and e) [19]. Altogether, *in vitro* cigarette smoke exposure mimicked the elevated expression of UCHL1 detected in airway epithelial cells obtained from smokers.

### Inhibition of UCHL1 represses TGF-β1-induced COL1A1 in lung epithelial cells

**Figure 4.**
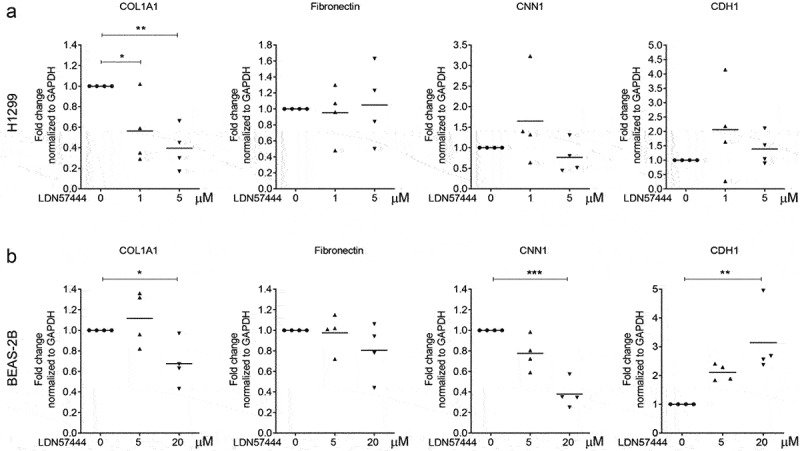
UCHL1-specific inhibitor suppresses expression of COL1A1. (a, b) UCHL1 inhibitor represses the expression of COL1A1 in H1299 (a) or BEAS-2B (b) cells. Both cell lines were sham-exposed or exposed with LDN57444, a UCHL1 inhibitor, when the cells were 80% confluent. The control group was treated with equal volumes of DMSO. Cells were harvested with TRIzol at 24 h post treatment. The mRNA expression of COL1A1, fibronectin, CNN1, and CDH1 were determined by real-time qRT-PCR (n = 4). Statistical significance was determined using two-tailed unpaired *t*-test, *p < 0.05, **p < 0.01, ***p < 0.001.

We previously reported that UCHL1 is a crucial modulator of epithelial–mesenchymal transition (EMT) [20]. To further validate a relation between UCHL1 and EMT markers, co-expression patterns between UCHL1 mRNA expression and potential EMT downstream genes were retrieved from the Cancer Cell Line Encyclopaedia [21], sourced from the TCGA provisional datasets hosted at cBioPortal [22,23]. These data indicate that the transcript level of ECM-related genes (including COL1A1, COL3A1, fibronectin, CNN1, CDH1, and CDH2) instead of EMT markers are closely associated with the expression of UCHL1 (Supplemental Figure 3A and B). To further investigate the functional association between UCHL1 and these genes, we used LDN57444 to inhibit the enzymatic activity of UCHL1 [24]. Human lung cancer cell line H1299 and human immortalized bronchial epithelial cell line BEAS-2B were exposed to LDN57444 and the expression of several ECM marker genes was assessed. Intriguingly, upon LDN57444 treatment, the expression of COL1A1 was decreased significantly both in H1299 (Figure 4a) and BEAS-2B (Figure 4b) cells. The expression of CNN1, a basic smooth muscle protein, was repressed by up to 40% upon LDN57444 treatment in BEAS-2B cells (Figure 4b), but not in H1299 (Figure 4a). Expression of the epithelial cell marker CDH1 was increased with the increasing dose of LDN57444 in BEAS-2B, but not in H1299 cells. In addition, the expression of COL1A1 was measured in primary bronchial epithelial cells exposed to different doses of CSE. To our surprise, a decrease of COL1A1 expression was observed upon the stimulation of CSE (Supplemental Figure 4).

**Figure 5.**
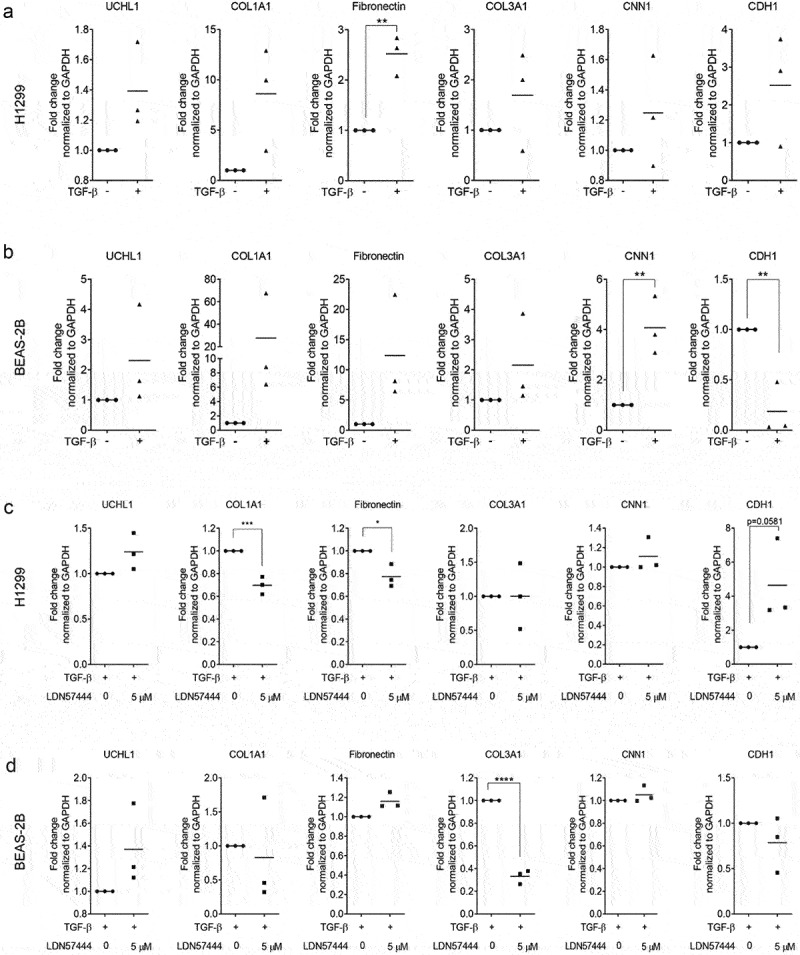
UCHL1 inhibitor represses TGF-β1-induced upregulation of COL1A1. (a, b) H1299 cells (a) or BEAS-2B cells (b) were incubated with medium containing 0.5% serum and 50 ng/ml Vitamin C for 12 h when the cells were 80% confluence. Cells were treated with 10 ng/ml TGF-β1 for 72 h (medium containing TGF-β1 was changed every 24 h). (c, d) H1299 cells (c) or BEAS-2B cells (d) were incubated with medium containing 0.5% serum and 50 ng/ml Vitamin C for 12 h. Cells were treated with 10 ng/ml TGF-β1 for 48 h (medium containing TGF-β1 was changed every 24 h), and co-treated with TGF-β1 and 5 µM LDN57444 or DMSO for another 24 h. Cells were harvested for RNA isolation and the mRNA expression of COL1A1, fibronectin, COL3A1, CNN1, and CDH1 were performed by real-time qRT-PCR (n = 3). Statistical significance was determined using two-tailed unpaired t-test, *p < 0.05, **p < 0.01, ***p < 0.001, ****p < 0.0001.

As TGF-β1 is known to induce the synthesis and secretion of ECM proteins including collagens [25], we next tested whether inhibition of UCHL1 activity could affect TGF-β1 induced-expression of ECM genes. As shown in Figure 5a and b, the expression of COL1A1 increased 8.6-fold upon treatment with TGF-β1 in H1299 (*p* = 0.061), but not in BEAS-2B cells (27.6-fold, *p* = 0.25). TGF-β1 upregulated fibronectin 2.5-fold (*p* = 0.0025) in the H1299 cell line and 12.3-fold in BEAS-2B cells (*p* = 0.089) (Figure 5a and b). TGF-β1 did not upregulate UCHL1 expression in these two cell lines (Figure 5a and b), whereas it did induce the expression of CNN1 and inhibited the expression of CDH1 in BEAS-2B cells (Figure 5b). Intriguingly, UCHL1 inhibitor LDN57444 prevented the TGF-β1-induced expression of COL1A1 and fibronectin (*p* < 0.005 and *p* = 0.017, respectively) and slightly induced the expression of CDH1 (*p* = 0.058) in H1299 cells (Figure 5c), compared with TGF-β1 only. This was not observed in BEAS-2B (Figure 5d). A pronounced decreased expression of COL3A1 was found in BEAS-2B cells upon co-treatment of LDN57444 and TGF-β1 *p* < 0.0001 (Figure 5d) even though no significant TGF-β1-induced upregulation was observed (Figure 5b). Overall, UCHL1 seems to act as a mediator between TGF-β1 and ECM genes COL1A1 and fibronectin.

### COL1A1 expression is increased as a response to targeted endogenous upregulation of UCHL1

**Figure 6.**
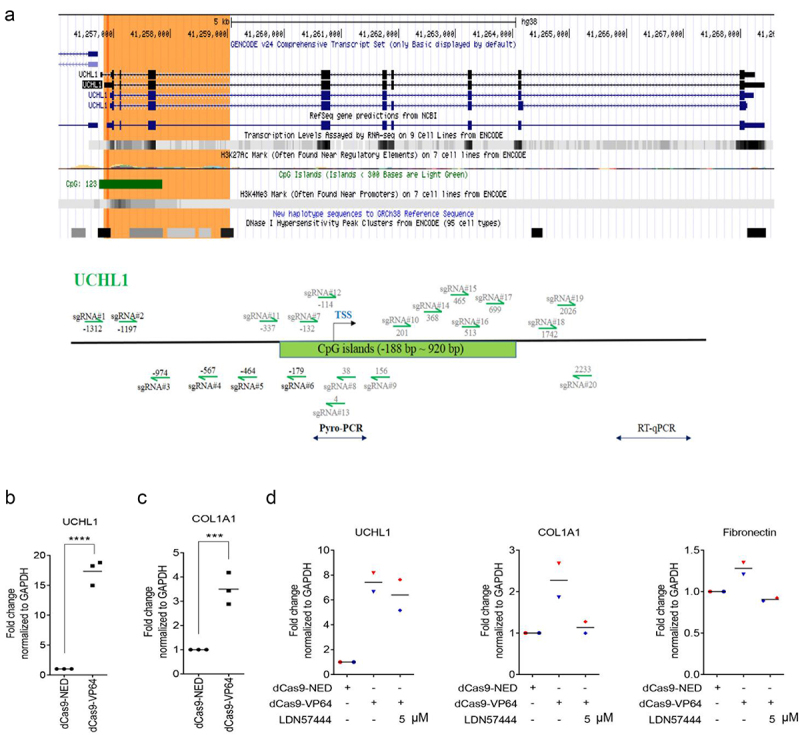
COL1A1 is increased as a response to targeted endogenous upregulation of UCHL1. (a) Schematic structure of the UCHL1 promoter. The green highlighted part shows the CpG island and the sgRNAs designed around the TSS are indicated as arrows. (b, c) Targeting dCas9-VP64 induced upregulation of UCHL1 and further increased the expression of COL1A1. BEAS-2B cells were seeded and transiently co-transfected with 1 µg a mixture of UCHL1 sgRNA (#4/5/6) together with 1 µg dCas9-NED-mCherry, and 1 µg dCas9-NED or dCas9-VP64 through PEI (n = 3). (d) The transfection is same as (b), 24 h post transfection, cells were treated without or with 5 µM LDN57444 for 24 h (n = 2). The control group was treated with equal volume of DMSO. mCherry-positive cells were collected and sorted by FACS at 48 h post treatment. Total RNA was isolated and real-time qRT-PCR was performed to measure gene expression. Statistical significance was determined using two-tailed unpaired t-test, *p < 0.05, **p < 0.01, ***p < 0.001, ****p < 0.0001.

To further explore the function of UCHL1 in lung epithelium and to validate the role of UCHL1 in the regulation of ECM markers, sgRNAs targeting the UCHL1 TSS were designed, as indicated in Figure 6a. Various sgRNA combinations were screened for effective upregulation by co-transfecting with plasmids encoding dCas9-VP64 (artificial transcription activator) in BEAS-2B and 16HBE cells, which show relatively low expression of UCHL1 (Supplemental Figure 5A and B). The combination of three sgRNA (#4/5/6) showed 4.8-fold induction of UCHL1 expression in BEAS-2B cells. To circumvent the effect of low transfection efficiency of the large dCas9 plasmid, cells stably expressing dCas9-VP64 were created. Despite the high dCas9-VP64 expression (100 times higher in stable cells compared to transiently transfected cells), the combination of sgRNAs upregulated UCHL1 expression again 5.1-fold (ranging from 3.6- to 7.9-fold; Supplemental Figure 5C and D).

Next, to increase number of effector domains with the MS2 system [26], BEAS-2B cells stably expressing dCas9-NED or dCas9-VP64 were transfected with MS2 plasmids and the sgRNA combination (Supplemental Figure 5E): Compared to MS2-NED, MS2-p65-HSF (a fusion protein consisting of MS2, p65 (the trans-activation subunit of NF-kB) and HSF1 (the activation domain of human heat shock factor 1)) induced a 3.8-fold upregulation of UCHL1 in the dCas9-NED cells, confirming effective MS2 binding to sgRNAs (p = 0.0044). For the dCas9-VP64 expressing cells, however, co-expressing MS2-p65-HSF did not further improve the induction of UCHL1 expression; neither increasing the relative amount of the MS2-effector plasmids, increasing incubation time (to day 6), nor selection of transfected cells using hygromycin could consistently improve the induction of UCHL1 expression (Supplemental Figure 5E).

As an alternative to antibiotic resistance selection, sorting based on fluorescence (tags or co-transfection of reporter plasmids) was next exploited to improve the reliability of the readouts. Towards this end, BEAS-2B cells were transiently co-transfected with dCas9-NED-mCherry or a GFP reporter plasmid and sorting was performed to enrich the transfected cells. Although dCas9-NED is likely to compete with dCas9-VP64 for binding to the UCHL1 promoter, blocking of upregulation was not observed in bulk cells (2.8-fold induction when co-transfected with GFP versus 2.2-fold when co-transfected with dCas9-NED-mCherry). Upon sorting, the upregulation effect was clearly increased: for dCas9-VP64, a 17-fold induction of UCHL1 expression was observed when co-transfected and sorted for mCherry (Figure 6b and Supplemental Figure 5 F). When co-transfected with a separate GFP plasmid, sorting resulted only in a 7.2-fold induction in UCHL1 expression in GFP positive cells, even though these cells showed the highest increase in the bulk population (Supplemental Figure 5 F). Also for a lung epithelial cancer cell line (H1299), similar upregulation was achieved for bulk cells, although upon sorting, the improvement on detecting UCHL1 upregulation was more modest (ca 5.8–fold for mCherry-positive cells and 2.2-fold for GFP-positive cells) (Supplemental Figure 5 G). In addition, the combination of PRDM9 (writing H3K4me3) and Dot1l (writing H3K79me) also induced around 4-fold upregulation of UCHL1 expression upon mCherry sorting, compared with NED control or PRDM9 mutant and Dot1l mutant (Supplemental Figure 5 H).

Importantly, a 3.5-fold induction of COL1A1 mRNA expression was observed in BEAS-2B upon the dCas9-VP64-induced UCHL1 activation (Figure 6c), although no such induced effects were found on the expression of fibronectin or other ECM-related genes (Supplemental Figure 6). Exogenous UCHL1 overexpression from cDNA plasmid resulted in dramatic upregulation of UCHL1 in H1299 and BEAS-2B cells (95 to 279-fold and 217 to 675-fold, respectively, Supplemental Figure 7, A‒D). Surprisingly, no clear induction of COL1A1 or other ECM-related genes was obtained in either H1299 or BEAS-2B cells upon exogenously overexpressing UCHL1. However, the exogenous upregulation of UCHL1 did induce the expression of CNN1, especially in H1299 cells (2.2-fold, Supplemental Figure 7A), and stimulate the expression of LAMA1 in BEAS-2B cells (1.6-fold, Supplemental Figure 7D). To further confirm the association between UCHL1 and COL1A1, cells were treated with the inhibitor LDN57444 during the dCas9-VP64 targeted upregulation of UCHL1. Consistent with Figure 6c, the expression of COL1A1 increased about 2.5-fold with the upregulation of UCHL1 (7.4-fold), but this effect was almost completely prevented by 5 µM LDN57444. A similar effect was observed for the expression of fibronectin (Figure 6d).

### Downregulation of UCHL1 by epigenetic editing suppresses the expression of COL1A1 and fibronectin

**Figure 7.**
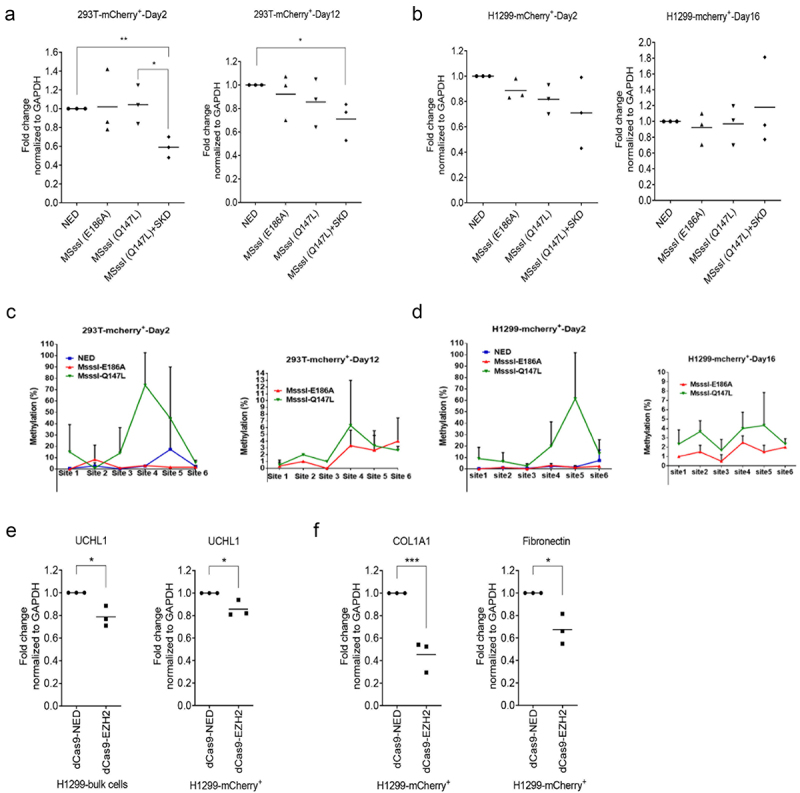
Downregulation of UCHL1 by epigenetic editing suppresses the expression of COL1A1 and fibronectin. (a, b). DNA methyltransferase tools to downregulate UCHL1. HEK293T (a) and H1299 (b) cells were cotransfected with 1 µg UCHL1 sgRNA (#1/2/3) and 2 µg (in total) dCas9-mcherry-ED(s). For mCherry-based enrichment, cells were collected 60 h after transfection and dCas9-mCherry-ED expressing cells were sorted by FACS. Half of the sorted cells were harvested for short-term (day 2) expression measurements and the remaining cells were seeded in 24-well plates for long-term expression (day 12 for HEK293T, day 16 for H1299). (c, d). same as (A, B), genomic DNA at day 2 (left panel) and day 12/16 (right panel) was isolated from HEK293T (A) or H1299 (B) sorted cells, simultaneously with total RNA isolation, and used for pyrosequencing. dCas9-MSssI (E186A)-mCherry (inactive mutant); dCas9-MSssI (Q147L)-mCherry (active mutant). (e, f). H1299 cells were co-transfected with 1 µg a mixture of UCHL1 sgRNA (#1/2/3) and 2 µg dCas9-EZH2-mCherry or dCas9-NED-mCherry by PEI. Cells were collected 48 h post transfection, and mCherry-positive cells were sorted by FACS. The mRNA expression of UCHL1 (e), COL1A1, and fibronectin (f) were analysed by real-time qRT-PCR (n = 3). Significance was analysed by one-way ANOVA, *p < 0.05, **p < 0.

As UCHL1 is frequently repressed by aberrant DNA methylation in a range of carcinomas, we wondered whether specific DNA methylation might result in efficient and sustained downregulation of UCHL1, as reported for other genes [27–29]. To build on the observed improvement of readout by sorting cells, various variants of the DNA methyltransferase MSssI fused to dCas9 with mCherry tag were constructed and tested in mammalian cells for targeted downregulation of UCHL1. HEK293T and H1299 cells were co-transfected with plasmids transiently expressing dCas9-NED-mCherry, dCas9-MSssI (E186A)-mCherry, dCas9-MSssI (Q147L)-mCherry or combination of dCas9-MSssI (Q147L)-mCherry and dCas9-SKD (without mCherry) together with combinations of plasmids expressing the UCHL1 sgRNA (#1/2/3) for which downregulation was observed in BEAS-2B cells upon targeting SKD (data not shown). No difference in UCHL1 gene expression levels was observed on day 2 after transfection in the bulk population for either effector domain (combination) (Supplemental Figure 8). However, in the HEK293T sorted population, a 40% decrease in gene expression was observed for the combination of dCas9-MSssI (Q147L) and dCas9-SKD, compared to the dCas9-NED control. This decrease in gene expression was not observed for cells expressing only dCas9-MSssI (Q147L) in the sorted population (Figure 7a). Next, we analysed the sustained effect of the effectors in the sorted population at day 12 post transfection. Again, no effect of MSssI (Q147L) was detected, but the repressive effect was sustained, albeit less pronounced, for the combination of MSssI (Q147L) and SKD (Figure 7a).

In the bulk population of H1299, the targeting of a larger effector domain to the gene seemed to affect the gene expression, as the methylation defective dCas9-MSssI (E186A) (no methylation activity, but it has higher DNA binding ability) variant (around 1.2 kb in size) repressed expression to 80%, while dCas9-SKD (0.2 kb) and dCas9-MSssI (Q147L) (active mutant) did not result in repression (Supplemental Figure 8). Sorting of the mCherry positive H1299 cells, did not result in an improved repression of UCHL1. As expected for a catalytically inactive effector domain, upon long-term culturing of the sorted cells, gene expression levels of dCas9-MSssI (E186A) treated cells went back to the expression level of dCas9-NED treated cells (Figure 7b). However, also no repression was observed after sorting or after subculturing of the sorted H1299 population for dCas9-MSssI (Q147L) with or without dCas9-SKD.

To determine the methylation status of the UCHL1 promoter region after treatment with dCas9-MSssI (Q147L), pyrosequencing was performed for the region around the TSS in both the HEK293T and H1299 sorted populations. A significant increase in methylation was observed at day 2 post transfection with dCas9-MSssI (Q147L) at CpG sites #4 and #5: ca 70% at site #4 (12 bp upstream of TSS) for HEK293T and ca 60% at site #5 (6 bp upstream of TSS) for H1299 (Figure 7c and d), even though no gene repression was observed in H1299 cells. This indicates that although methylation was induced at the TSS, which is at a 1 kb distance from the targeted site, no subsequent effect on gene expression was observed. The DNA methylation induced by MSssI (Q147L) decreased to 6.3% at site #4 for HEK293T and to 4.3% at site #5 for H1299 after 12 days, indicating that the induced DNA methylation was not sustained, even though repression of UCHL1 was still present in HEK293T cells. To target DNA methylation to the CG island of UCHL1, sgRNA (#6/9/11/14), ranging from 337 bp upstream and 368 bp downstream of TSS, were co-transfected with dCas9-MSssI variants and dCas9-SKD into H1299 and HEK293T cells, but no repression below 50% of UCHL1 expression in sorted HEK293T or H1299 was induced (Supplemental Figure 9A‒C).

Targeting histone methyltransferase EZH2 (SET domain) fused to dCas9-mCherry to UCHL1 resulted in around 20% downregulation of UCHL1 expression at 48 h post transfection both in bulk and in the sorted population of H1299 cells (*p* = 0.015 and *p* = 0.027, respectively) (Figure 7e). For H1299 cells, this repression in UCHL1 expression was associated with around 50% repression of COL1A1 and 30% repression of fibronectin (Figure 7f and Supplemental Figure 10). No repression of UCHL1 was obtained with the same experimental setup in HEK293T cells despite their higher transfection efficiency than H1299 (Supplemental Figure 11A). No persistent downregulation of UCHL1 was achieved in H1299 (16 days) or HEK293T (12 days) cells after transient expression of EZH2 (Supplemental Figure 11B and C).

## Discussion

In this study, we confirmed the high expression of UCHL1 in the airway epithelium of current smokers, and found a negative correlation between transcription of the UCHL1 gene and DNA methylation at specific CpG sites in these cells from the same individuals. We established targeted repression of UCHL1 expression in the lung cancer cell line H1299 and showed that UCHL1 directs the expression of the extracellular matrix genes COL1A1 and fibronectin.

Our observation that smokers have more UCHL1 expressing cells in the airways, supports previous reports showing that UCHL1 was expressed at higher level in current smokers [2,3,6]. In the study by Carolan *et al.*, UCHL1 expression was only evident in neuroendocrine cells of the airway epithelium in non-smokers, whereas in smokers, it was also expressed in ciliated epithelial cells. In the context that UCHL1 is involved in the degradation of unwanted, misfolded, or damaged proteins within the cell and is overexpressed in >50% of lung cancers, this observation made the authors to suggest that overexpression of UCHL1 in chronic smokers may represent an early event in the complex transformation from normal epithelium to overt malignancy [2]. Boelens *et al*. found high expression of UCHL1 in smokers, which was higher in malignant transformed epithelial cells of squamous cell lung carcinoma (SCC), which would be consistent with expression in either airway basal cells or metaplastic squamous cells, but not with a ciliated cell origin [6].

In the current study, we were interested in the epigenetic regulation of UCHL1 expression and found that UCHL1 expression was inversely correlated with DNA methylation at two CpG sites around the TSS in lung epithelium. This negative correlation is in accordance with six lung cell lines detected and data obtained for various cancer types that addressed regions containing these two CpG sites [4,30–34]. In primary nasopharyngeal tumours, methylation in the UCHL1 promoter region containing these two CpG sites was much higher than in normal tissues [4] and a similar observation was made in oesophageal squamous cell carcinoma primary tumours [31]. Despite the inverse correlation for two CpG sites in cell lines, differential DNA methylation of UCHL1 was only detected for CpG 6 in non-smokers and ex-smokers. In contrast, short term (24 h) CSE exposure did not significantly downregulate methylation of UCHL1 at CpG sites#1–6. This indicates that the observed decrease in methylation of CpG 6 in LCM samples of (ex)smokers can only be observed after long-term smoking and in the presence of other cell types in the airways, including inflammatory cells, secreting mediators. However, another study reported that in oral squamous cell carcinoma (OSCC), promoter methylation of UCHL1 was higher in OSCC tumours of smokers compared with non-tumour smokers. Their data also showed that UCHL1 methylation increased markedly after 10–15 days of exposure to cigarette smoke condensates in DOK cells (an oral keratinocyte from a heavy smoker with OSCC) through methylation-specific PCR (MSP) [35]. The different effects of smoking on DNA methylation found in other studies compared to our results could be explained by a difference in tissues and cells analysed, the different range of CpGs covered and a different readout technology used (MSP versus pyrosequencing).

Although CSE exposure of PBECs did not change DNA methylation of UCHL1, it did increase the expression of UCHL1. This is consistent with two datasets from the GEO database (GSE30660 and GSE82137), showing an upregulated UCHL1 expression in PBECs exposed to gaseous cigarette smoke using the ALI cell culture model. These results are also in line with data obtained from BEAS-2B cells exposed to beeswax pellets containing cigarette smoke condensate [36]. Importantly, compared with CSE exposure only, the induction of UCHL1 expression was maintained at least 24 h upon the removal of CSE, which indicates that the high expression of UCHL1 induced by cigarette smoke was maintained in the absence of CSE. CSE unexpectedly did not induce COL1A1 mRNA expression as was shown in a previous study by Milara et al. [37]. In that study, not only exposure of PBECs to 2.5% CSE did induce COL1A1 expression, they also showed that in PBECs obtained from small bronchi of smokers with and without COPD, COL1A1 protein expression was shown to be inversely correlated with FEV1 (% of predicted). Collectively, the effect of CSE on ECM protein expression is closely correlated with specific cell types and the molecular mechanism of UCHL1 affecting the transcriptional expression of ECM genes needs further exploration.

As smoking is closely associated with various airway diseases, in addition with pulmonary fibrosis, UCHL1 expression in epithelium might provide a molecular link between smoking and airway submucosal fibrosis. With respect to a possible function of UCHL1 in ciliated cells in lung airway epithelium [2], for comparison, when looking to other cell types, UCHL1 is also highly expressed in liver myofibroblasts [13], cultured dermal fibroblasts from colorectal cancer [38] and human cutaneous wounds [39]. In these cell types, treatment with TGF-β1 induced upregulation of UCHL1, which was not found in our lung epithelial cells, suggesting that the induction of UCHL1 occurs in a cell-type specific manner and that UCHL1 may have a different role in different cell types. In order to investigate a possible functional effect of UCHL1 on ECM remodelling in lung epithelial cells, a UCHL1-specific inhibitor LDN57444 was used. Application of LDN57444 revealed that the expression of COL1A1 in two epithelial cell lines can be significantly repressed by UCHL1 inhibitor treatment. As TGF-β1 is also a well-known stimulator for ECM production [40], and as CSE did not induce COL1A1 in our epithelial cells, we subsequently checked whether TGF-β1-induced COL1A1 and fibronectin could be repressed by the UCHL1 inhibitor. The expression of both COL1A1 and fibronectin decreased upon co-treatment of TGF-β1 and LDN57444, but this phenomenon was only observed in the lung cancer epithelial cell line H1299, and not in the human normal lung epithelial cell line BEAS-2B. In the lung, the source of TGF-β1 may be from different cell types (e. g. fibroblasts, macrophages, and epithelial cells), and it is very likely that *in vivo*, both CSE and TGF-β1 at the same time contribute to the role that UCHL1 plays in the regulation of extracellular matrix genes in airway epithelium.

To further explore a possible pro-fibrotic function of UCHL1 in epithelial cells, we explored CRISPR/dCas9-based gene expression modulation. By targeting dCas9-VP64, UCHL1 expression was induced 17-fold in BEAS-2B cells. Interestingly, upregulation of COL1A1 as a response to endogenous upregulation of UCHL1 by VP64 was found, whereas no clear induction of COL1A1 was observed after exogenously overexpressing UCHL1 with a 100- to 400-fold upregulation. As UCHL1 has alternative splicing variants and potentially different TSS [41], this might be an explanation for the difference in cDNA-encoded ectopic overexpression *versus* endogenous induction of UCHL1 expression. Importantly, the UCHL1-mediated induction of COL1A1 and fibronectin was inhibited by the UCHL1 inhibitor, indicating that any aspecific effects of CRISPR platform cannot explain this observation.

We then set out to induce efficient silencing of UCHL1 by targeting dCas-EZH2, as reported for other genes [42,43]. EZH2, an enzymatically active core subunit of polycomb repressive complex 2 (PRC2), methylates lysine 27 of histone H3 (H3K27) to induce chromatin compaction, thereby contributing to potentially mitotically stable transcriptional silencing [44]. In our studies, repression of UCHL1 expression and subsequent inhibition of COL1A1 and fibronectin mRNA expression was observed upon endogenous downregulation of UCHL1 via dCas9-EZH2. Our data suggest that UCHL1 can be an important player in the synthesis of extracellular matrix, although then most likely in lung cancer and not yet clear in normal (airway) epithelium. Lei *et al*. recently showed that both inhibition and knockdown of UCHL1 also in mice cardiac fibroblasts suppressed TGF-β1-induced upregulation of COL1A1 protein expression [16], which is in agreement with our results. In addition, downregulated fibronectin expression as a result of siRNA-suppressed UCHL1 was previously reported in HEK293T and C33A cells [45].

Although epigenetic editing bears the promise to induce sustained effects [46–50], the transcription repression of UCHL1 did not persist after transient expression of dCas9-EZH2 construct in H1299 cells. Consistently, acquisition of histone methylation alone is not enough for stably silencing the transcriptional expression of UCHL1, as also reported for, e.g., HER2, for which persistent silencing required both a DNA methyltransferase and EZH2 [46,51]. Although not in H1299 cells, cotargeting of dCas-MSssI (Q147L) and dCas-SKD did result in prolonged downregulation of UCHL1 in HEK293T cells, confirming other reports [17,27,29]. Straightforward long-term reprogramming of gene expression by epigenetic editing would provide a powerful and flexible approach to study the role of any given gene in various contexts.

In summary, we confirmed that the expression of UCHL1 in airway epithelium can be induced by cigarette smoke and that UCHL1 transcription was negatively associated with the endogenous methylation status of the UCHL1 promoter. Besides this, in an *in vitro* model, the expression of ECM genes, especially COL1A1 and fibronectin, responded to gain-of-function and loss-of-function of UCHL1 mediated by CRISPR/dCas9-based expression modulation. The effects of induced UCHL1 could be prevented by a UCHL1 inhibitor, pointing to a possible therapeutic strategy in the future, in diseases where UCHL1 can be expected to be the main player. Altogether, we provide additional insight into the role of UCHL1 in the regulation of extracellular matrix genes in epithelial cells. **UCHL1 plays a role in the regulation of extracellular matrix genes in epithelial cells where possibly both CSE and TGF-β1 contribute *in vivo.*** Still there is likely other functionality of UCHL1 in the normal airway epithelium, which is yet to be explored further.

## Materials and methods

### Patient characteristics

Lung tissue was obtained from individuals during thoracotomy who had a lung function that was in normal range. For each individual, the number of pack-years (number of packs of cigarettes smoked per day × years of consumption) and current smoking status was assessed. Individuals were divided into never-smokers (n = 10), ex‐smokers (n = 14), who quitted smoking at least 2 years before surgery, or current smokers (n = 13), who continued smoking until surgery.

PBECs were obtained from tracheal (or main stem bronchial) tissue collected from lung transplant donors post-mortem, within 8 h after transplantation while using the selection criteria for transplant donors according to the Euro transplant guidelines. No information was available from the transplant donors. The study protocol was consistent with the Research Code of the UMCG (https://www.umcg.nl/SiteCollectionDocuments/English/Researchcode/umcg-research-code-2018-en.pdf) and the national ethical and professional guidelines (‘Human tissue and medical research: code of conduct for responsible use (2011),’ https://www.coreon.org/wp-content/uploads/2020/04/coreon-code-of-conduct-english.pdf). The patient characteristics are described in Table 2.

**Table 2.** Characteristics of subjects and experimental design.

	Age (years)	Gender	Smoking status	Pack years	FEV1%pred	FEV1/FVC%	IHC	LCM
Sample 1	63	f	ns	0	95	84	+	+
Sample 2	49	m	ns	0	78.5	78	+	+
Sample 3	50	m	ns	0	94	78	+	+
Sample 4	45	m	ns	0	80	73	+	+
Sample 5	52	f	ns	0	121	83	+	+
Sample 6	72	f	ns	0	91	81	+	+
Sample 7	45	m	ns	0	130	83	+	-
Sample 8	69	f	ns	0	78.2	85.8	+	-
Sample 9	65	f	ns	0	100	73	+	-
Sample 10	67	f	ns	0	114.4	80.3	+	-
Sample 11	45	f	cs	30	103.6	81.8	+	+
Sample 12	49	f	cs	33	87.3	82.3	-	+
Sample 13	43	m	cs	NA	102	76	+	+
Sample 14	64	m	cs	40	116	78	+	+
Sample 15	52	f	cs	20	81.6	74.2	+	+
Sample 16	55	f	cs	32	103	77	+	+
Sample 17	63	f	cs	NA	96.0	75.3	+	-
Sample 18	47	f	cs	50	61	75	+	-
Sample 19	54	f	cs	38	103.8	90.1	+	-
Sample 20	52	f	cs	40	101.2	78.2	+	-
Sample 21	65	f	cs	NA	88.6	62.4	+	-
Sample 22	53	m	cs	60	90	70	+	-
Sample 23	66	m	cs	40	102	72	+	-
Sample 24	74	m	es	53	99.9	71.4	+	+
Sample 25	63	m	es	65	64	79	+	+
Sample 26	67	m	es	34	71.7	69.7	-	+
Sample 27	59	f	es	35	78	79	+	+
Sample 28	69	m	es	20	104	70	+	+
Sample 29	60	m	es	NA	NA	NA	-	+
Sample 30	68	m	es	45	49.3	77.7	+	-
Sample 31	65	f	es	11.5	90.4	75.8	+	-
Sample 32	43	f	es	1.5	121	75	+	-
Sample 33	66	m	es	10	72.4	78.0	+	-
Sample 34	50	m	es	NA	104.9	86.6	+	-
Sample 35	71	m	es	NA	NA	80.2	+	-
Sample 36	62	m	es	NA	99	79	+	-
Sample 37	68	f	es	51	100	73.6	+	-

### Culture of primary bronchial epithelial cells (PBECs)

PBECs were cultured with keratinocyte serum-free medium (KSFM, Gibco) containing 0.2 ng/ml epidermal growth factor (EGF) and 25 µg/ml pituitary bovine extract (BPE) supplemented with 1 µM isoproterenol, until confluence on fibronectin/collagen pre-coated plates.

### Cell culture

Human bronchial epithelial cells BEAS-2B (ATCC CRL-9609) and human lung cancer cells H1299 (ATCC CRL-5803) were cultured in RPMI (Gibco, UK). Human bronchial epithelial cells 16HBE were cultured in Eagle minimum essential medium (EMEM, Lonza, BioWhittaker, USA) on plates pre-coated with 10 µg/ml BSA and 33 µg/ml Collagen. All cell line culture media were supplemented with 10% foetal bovine serum (FBS, Sigma, France), 2 mM L-glutamine (Lonza, BioWhittaker, Belgium), and 1% penicillin–streptomycin (Gibco, Grand Island, USA). Cells were cultured in a humidified atmosphere at 37°C supplemented with 5% CO_2_. All cell lines were tested for mycoplasma contamination.

### Stimulation of epithelial cells

For CSE exposure, PBECs were seeded into pre-coated 12-well plates (1.5 × 10^5^ cells per well) and treated with 0–20% freshly prepared CSE for 4 h, 8 h, and 24 h when the cells reached 80% confluence. CSE was prepared as previously described with some modifications [52]. Briefly, mainstream smoke derived from two 3R4F Kentucky Research cigarettes (The Tobacco Research Institute, University of Kentucky, Lexington, KY, USA) was bubbled slowly into 20 mL of medium (100% CSE). The extract was prepared freshly, diluted with cell culture medium to the required concentrations, and used within 30 min.

For transforming growth factor (TGF) -β1 stimulation, BEAS-2B and H1299 cells were seeded in 12-wells plates (1.5 × 10^5^ cells per well) and 24 h later, the medium was replaced with a medium containing 0.5% FBS and 50 ng/ml L-ascorbic acid 2-phosphate sesquimagnesium salt hydrate (vitamin C, Sigma-Aldrich) overnight. Subsequently, the cells were treated with 10 ng/ml recombinant TGF-β1 (Peprotech) for 72 h. The medium containing TGF-β1 was changed every 24 h, as described by Hosper et al. [25].

For UCHL1 inhibitor LDN57444 treatment, BEAS-2B and H1299 cells were seeded in 12-wells plates (1.5 × 10^5^ cells per well). 24 h later, cells were exposed to 0–20 µM LDN57444 (Sigma-Aldrich) or equal volume of vehicle (DMSO) for 24 h. For co-treatment with TGF-β1 and LDN57444, BEAS-2B and H1299 cells were seeded into 12-well plates (1.5 × 10^5^ cells per well), and 24 h later the medium was replaced with a medium containing 0.5% FBS and 50 ng/ml vitamin C overnight. Subsequently, cells were treated with 10 ng/ml TGF-β1 for 48 h with daily medium replacement, and co-treated with 10 ng/ml TGF-β1 and 5 µM LDN57444 for another 24 h. The control group was treated with TGF-β1 and an equal amount of DMSO. Cells were harvested for RNA isolation.

### Plasmids

Plasmid MLM3636, expressing single-guide RNAs in mammalian cells, was a kind gift from Keith Joung (Addgene plasmid # 43860). Plasmid pMLM2.0 was constructed from MLM3636 by replacing the sgRNA expression cassette with the sgRNA2.0 expression cassette of the lentiviral plasmid sgRNA (MS2)_zeo backbone (Addgene plasmid # 61427) [26]. Stretches of 20 bp sgRNA to target UCHL1 were designed using the online tool (http://crispr.mit.edu/). Double-stranded oligonucleotides containing these targeting sites (listed in Supplemental Table 2) were cloned into BsmBI-digested pMLM2.0.

The plasmid containing the gene of the mammalian codon-optimized dCas9-VP64 activator (a tetramer of the viral VP16 transcriptional activator) was a kind gift from Keith Joung (Addgene; pMLM3705, #47754). An additional multiple-cloning site was inserted by replacing the VP64 coding sequence in dCas9-VP64 with a sequence containing a PacI restriction site, the new plasmid was referred to as No Effector Domain, pdCas9-NED (Addgene #109358) [53].

Plasmids expressing C-terminally mCherry-tagged dCas9-MSssI (Q147L/E186A) in mammalian cells were constructed as follows [54]: to create in-frame fusion between dCas9-MSssI and the P2A-mCherry-tag, the double-stranded oligonucleotide AK473-AK474 (5’-CGCGCCCAT ATGTTAATTAACAATTAA/5’-CCGGTTAATTGTTAATTAACATATGGG) was inserted between the SgsI (AscI) and BshTI (AgeI) restriction sites of pSYC-187 (Addgene#74,794). Insertion of AK473-AK474 preserved the flanking restriction sites, introduced a unique PacI site (underlined) and an in-frame stop codon. To abolish the stop codon, a short oligo-duplex (AK481-AK482, 5’-TAAGGTACCGA/5’-CCGGTCGGTACCTTAAT) was cloned between the PacI and the BshTI (AgeI) sites to obtain the plasmid pMCS-P2A-mCherry. (MCS stands for a sequence containing several restriction sites.) The SgsI (AscI) and Eco105I (SnaBI) fragment encoding the ‘MCS-P2A-mChery’ fragment was excised from pMCS-P2A-mCherry and cloned between the SgsI (AscI) and MssI (PmeI) sites of pdCas9-NED. The resulting plasmid pM-dCas9-(NED)-P2A_mCherry encodes a dCas9-P2A-mCherry fusion. The coding sequences of the MSssI (Q147L) and MSssI (E186A) variants were inserted, on SgsI-PacI fragments, between the SgsI and PacI sites of pdCas9-(NED)-P2A_mCherry. The new plasmids named pM-dCas9-MsssI (Q147L)-P2A-mCherry and pM-dCas9-MsssI (E186A)-P2A-mCherry express the respective dCas9-MSssI variant carrying the self-cleavable mCherry-tag. The catalytic domain of H3K27 histone methyltransferase enhancer of zeste homolog 2 (EZH2) was amplified with overhangs containing AscI and PacI restriction sites by PCR from pdCas9-EZH2. The EZH2 catalytic domain was subcloned into the pM-dCas9-NED-P2A-mCherry plasmid to yield pM-dCas9-EZH2-P2A-mCherry. The P2A-mCherry coding sequence was from the plasmid pSYC-187 [55], which was a kind gift from Seok-Yong Choi (Addgene plasmid # 74794). The catalytic domain of PRDM9 and DOT1L from dCas9-PRDM9 and dCas9-DOT1L, constructed as previously described [47], were subcloned into the pM-dCas9-NED-P2A-mCherry plasmid.

UCHL1 full-length cDNA (669 bp) was amplified from BEAS-2B cells and inserted into pcDNA4/HisMaxA between the BamHI and XbaI sites to generate the pcDNA4-UCHL1. Structure of the recombinant plasmids was confirmed by sequencing.

### Transfection

BEAS-2B cells were seeded into 6-well plates (5 × 10^5^ cells per well), then transiently co-transfected with pMLM2.0 plasmids expressing the respective sgRNA mixture (total 1 µg) and plasmids expressing the chimeric Effector Domain protein (pM-dCas9-ED, total 1 µg), using PEI transfection reagents (#23966, Polysciences, Inc.). Control cells were transfected using the same amount of irrelevant DNA. Cells were harvested at 60 h post transfection. Unless stated otherwise, all transfections were performed in at least three independent biological replicates.

### Fluorescence-activated cell sorting (FACS)

For mCherry-based enrichment, cells were transfected as described above, and harvested at 60 h post transfection by trypsinization, and cell pellets were resuspended in DPBS containing 2% FBS after centrifugation. Transfected cells were collected by FACS using a SH800S cell sorter (Sony Biotechnology) according to the manufacturer’s instruction.

### Immunohistochemistry staining

Formalin-fixed and paraffin-embedded lung tissue sections were stained for UCHL1 expression. Briefly, slides were deparaffinized with xylene and ethanol, and antigen retrieval was done by incubation in citrate buffer in a microwave for 15 min. After cooling down to room temperature, slides were incubated in PBS containing 0.3% H_2_O_2_ (v/v) for 30 min. Slides were incubated with rabbit anti-UCHL1 antibody (HPA005993, Sigma-Aldrich) at 1:800 for 1 h, then in a second step with HRP-conjugated goat-anti-rabbit antibody (1:100, Dako) for 30 min, and finally, in a third step, with HRP-conjugated rabbit-anti-goat antibody (1:100, Dako) for 30 min, and visualized with 0.05% diaminobenzidine (DAB, Sigma-Aldrich). Nuclear counterstaining was visualized using haematoxylin for 5 min and slides were dehydrated with ethanol. UCHL1 expression was quantified by manually counting the number of epithelial cells with only strong expression in all airways, which were corrected for the length of all airways along the basal end of the airway epithelium, as assessed by Aperio ImageScope viewing software 11.2.0.780 (Aperio). The characteristics of subjects and experimental design are described in Table 2.

### Laser capture micro-dissection (LCM)

Frozen lung sections of 8 mm thickness and quickly counterstained with haematoxylin for 30 seconds, washed with diethylpyrocarbonate-treated H_2_O thrice and washed with 100% ethanol once before specimen collection using Laser Micro-dissection System LMD6500 (Leica Microsystems, Amsterdam, The Netherlands). By manual identification of airway epithelial cell lining, desired areas were selected at 20× magnification and captured into adhesive caps (Carl Zeiss GmbH, Jena, Germany). Tissue dissections were immediately stored at −80°C and applied for total RNA and genomic DNA isolation using the AllPrep DNA/RNA Mini Kit (Qiagen, Venlo, The Netherlands), according to the manufacturer’s protocol.

### Bisulphite conversion and pyrosequencing

For DNA methylation analysis of the target regions, genomic DNA from different lung cell lines was extracted with TRIzol according to the manufacturer’s protocol and genomic DNA from LCM-collected epithelium was isolated as described above. Subsequently, 500 ng of genomic DNA was bisulphite converted with the EZ DNA Methylation-Gold kit (Zymo Research, USA) following manufacturer’s instructions. Bisulphite converted DNA was amplified by PCR using Pyromark PCR kit (Qiagen, Germany) with gene-specific primers and pyrosequencing was performed with a specific sequencing primer (Table 3) on Pyromark Q24 MD pyrosequencer (Qiagen, Germany) following manufacturer’s protocols. Primers were designed to span the region – 82 bps to +70 bps relative to the TSS (according to UCSC website). The percentage of methylation at specific CpG sites was analysed using the Pyromark Q24 Software (Qiagen, Germany).

### Real-time quantitative reverse transcription-PCR (real-time qRT-PCR)

Total RNA from PBECs or cell lines was isolated using TRIzol reagent (Thermo Fisher Scientific, Carlsbad, USA) according to the manufacturer’s protocol and RNA from LCM-collected epithelium was extracted as described above. cDNA was synthesized with random hexamer primers using the Revertaid cDNA synthesis kit (Thermo Scientific Inc.). Expression of UCHL1, collagen type I alpha 1 (COL1A1), fibronectin, collagen type III alpha 1 (COL3A1), calponin 1 (CNN1), E-cadherin (CDH1), laminin alpha 1 (LAMA1), and GAPDH genes were quantified using an ABI ViiA7 real-time PCR system (Applied Biosystems, USA) with ABsolute qPCR SYBR Green (Thermo Scientific, Inc.). Gene-specific primers are listed in Table 3 and Supplemental Table 3. Three technical replicates of real-time qRT-PCR were done for each biological repeat. Fold changes in mRNA expression above the control (dCas9-NED transfected cells) were calculated by the cycle threshold (ΔΔCt) method after normalization to GAPDH expression, unless stated otherwise.

**Table 3.** Information of PCR and sequencing primers.

Primer	Sequence (5’-3’)	Application
UCHL1-Fw	TTCCTGTGGCACAATCGGAC	qRT-PCR primer for UCHL1
UCHL1-Rv	CATCTACCCGACATTGGCCTT	
COL1A1-Fw	GGGATTCCCTGGACCTAAAG	qRT-PCR primer for COL1A1
COL1A1-Rv	GGAACACCTCGCTCTCCA	
COL3A1-Fw	CTGGACCCCAGGGTCTTC	qRT-PCR primer for COL3A1
COL3A1-Rv	CATCTGATCCAGGGTTTCCA	
Fibronectin-Fw	TCAACTCACAGCTTCTCCAA	qRT-PCR primer for Fibronectin
Fibronectin-Rv	TTGATCCCAAACCAAATCTT	
CNN1-Fw	CCAACCATACACAGGTGCAG	qRT-PCR primer for CNN1
CNN1-Rv	TCACCTTGTTTCCTTTCGTCTT	
CDH1-Fw	CATTGCCACATACACTCTCTTCT	qRT-PCR primer for CDH1
CDH1-Rv	CGGTTACCGTGATCAAAATCTC	
GAPDH-Fw	CCACATCGCTCAGACACCAT	qRT-PCR primer for GAPDH
GAPDH-Rv	GCGCCCAATACGACCAAAT	
dCas9-Fw	AATGGCATCCGAGACAAGCA	qRT-PCR primer for dCas9
dCas9-Rv	TGTGCTCGTGAAGACTGTCC	
UCHL1-pyro-Fw	GGTTTTGTTTTTGTTTTTTTTGTATAGG	PCR and sequencing primer for UCHL1 pyrosequencing (lower cases reflect the universal primer, Y is the CpG sites tested, subscript number indicating the site) (Site #4 is SNP-rs577696101-C/G according to UCSC)
UCHL1-pyro-Rv	gggacaccgctgatcgtttaAATCTCCA-TCYACTTAAACTACATCTTC	
Pyroseq-sequencing primer	TTGTATAGGTTTTATAGTG	
Pyroseq-sequence to analyse	**Y_1_**GTTTGGT**Y_2_**GGY_3_GTTTTATAGTTGTAGTTTGGGY_4_GGTTTY_5_G TTAGTTGTTTTTY_6_GTTTTTTTTAGGTTATTTTTGTY_7_GGGYGTTTY GYGAAGATGTAGTTTAAGTYGATGGAGATT	

### Statistical analysis

Nonparametric Mann–Whitney U-tests and unpaired/paired two-tailed t-tests were used to compare conditions for statistical analysis as indicated. Bonferroni correction was used to adjust the *p-*value as described. Statistics were performed using GraphPad Prism 8 software (GraphPad Software). All *in vitro* experiments were performed at least in three biological replicates, unless stated otherwise. Data were considered to be statistically significant if *p* < 0.05.

## Supplementary Material

Supplemental Material

## Data Availability

The datasets used during the study are currently available from the corresponding authors on reasonable requests.
