# Supplementary material for: Targeted epigenetic silencing of UCHL1 expression suppresses collagen-1 production in human lung epithelial cells
Source: Epigenetics. 2023 Feb 22;18(1):2175522. doi: 10.1080/15592294.2023.2175522 (PMC9980648; doi:10.1080/15592294.2023.2175522)
Supplement: Supplemental Material [file KEPI_A_2175522_SM3308.docx]

**Supplemental Materials and Methods**

**Plasmid constructions**

Plasmid pMLM2.0 was constructed by replacing the sgRNA expression cassette of the plasmid MLM3636 with the sgRNA2.0 expression cassette taken from the plasmid lenti sgRNA (MS2) zeo backbone. The sgRNA expression cassette of MLM3636 was deleted by Acc65I digestion and circularisation of the plasmid backbone. The resulting vector was digested with NheI and BamHI and ligated to the gel-purified NheI-BamHI fragment obtained from lenti sgRNA (MS2) zeo backbone (Addgene#61427).

The plasmid pMS2-P65-HSF1-HygroR was constructed by replacing the BcuI-MssI fragment carrying the CMV promoter and the dCas9 gene in pMLM3705 with the NheI-Eco32I fragment of the plasmid lenti MS2-P65-HSF1_Hygro. To construct the plasmid pMS2-NED-HygroR, first the P65-HSF1 gene was deleted from pMS2-P65-HSF1-HygroR by BamHI-BsrGI double-digestion. The BsrGI-EcoRI fragment of pMS2-P65-HSF1-HygroR carrying the T2A-Hygro genes was cloned between the Acc65I and EcoRI sites of pUC18. Subsequently, the T2A-Hygro fragment was excised from pUC18, and was used to replace the BamHI-EcoRI fragment of pMS2-P65-HSF1-HygroR to obtain pMS2-NED-HygroR. Nucleotide sequences of the plasmids constructed in this work are available upon request.

**Generation of BEAS-2B Stable Cell Lines**

HEK293T cells were co-transfected with the lentivirus packaging vector pCMV-ΔR8.91 (gag-pol 2nd generation packaging plasmid) along with pCMV-VSV.G (envelope plasmid) and pHAGE EF1α-dCas9-VP64 (a tetramer of the viral VP16 transcriptional activator), a gift from Rene Maehr & Scot Wolfe (Addgene plasmid # 50918) using PEI. Virus-containing supernatant was collected at 48 h and 72 h after transfection, supplemented with 10% FBS and 10 µg /ml polybrene (Sigma, USA), and used to transduce BEAS-2B cells. Two days after transduction, cells were selected with 8 µg /ml puromycin for one week. Subsequently, the resulting stable cell lines were cultured in 1 µg /ml puromycin-supplemented medium.

**Supplemental Figure and legends**

**
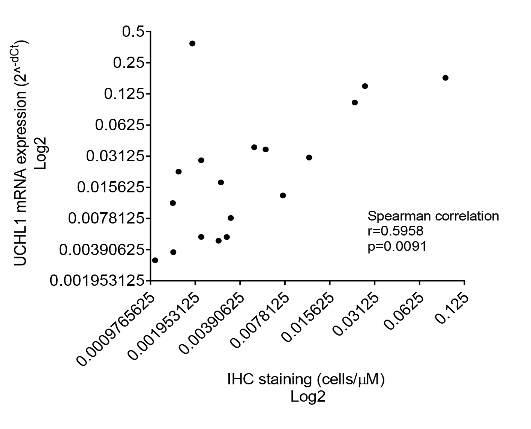
**

**Supplemental Figure 1.** **Correlation of mRNA and protein expression of UCHL1.** Lung airway epithelium from non-smoker, smoker, and ex-smoker was collected by LCM. mRNA expression of UCHL1 was measured by real-time qRT-PCR in Figure 1C. Immunohistochemical staining was performed to detect the protein expression of UCHL1 in the airway epithelium (Figure 1B) from the same patients as used for mRNA measurement. The characteristics of subjects are shown in Table 1. Correlation was established by nonparametric Spearman correlation test.





**Supplemental Figure 2. Correlation of mRNA expression and DNA methylation of UCHL1.** (A) DNA methylation status of 7 CpG sites in the promoter of UCHL1 was analyzed by pyrosequencing in different lung cell lines as indicated. (B) mRNA expression of UCHL1 in human lung cancer cells. (C) Correlation of mRNA expression and average DNA methylation level of UCHL1 was analyzed in LCM samples. The average methylation percentages of seven CpGs were detected by pyrosequencing (Figure 2B) and mRNA expression of UCHL1 was measured by real-time qRT-PCR (Figure 1C) from the same samples. Correlation was established by nonparametric Spearman correlation test.


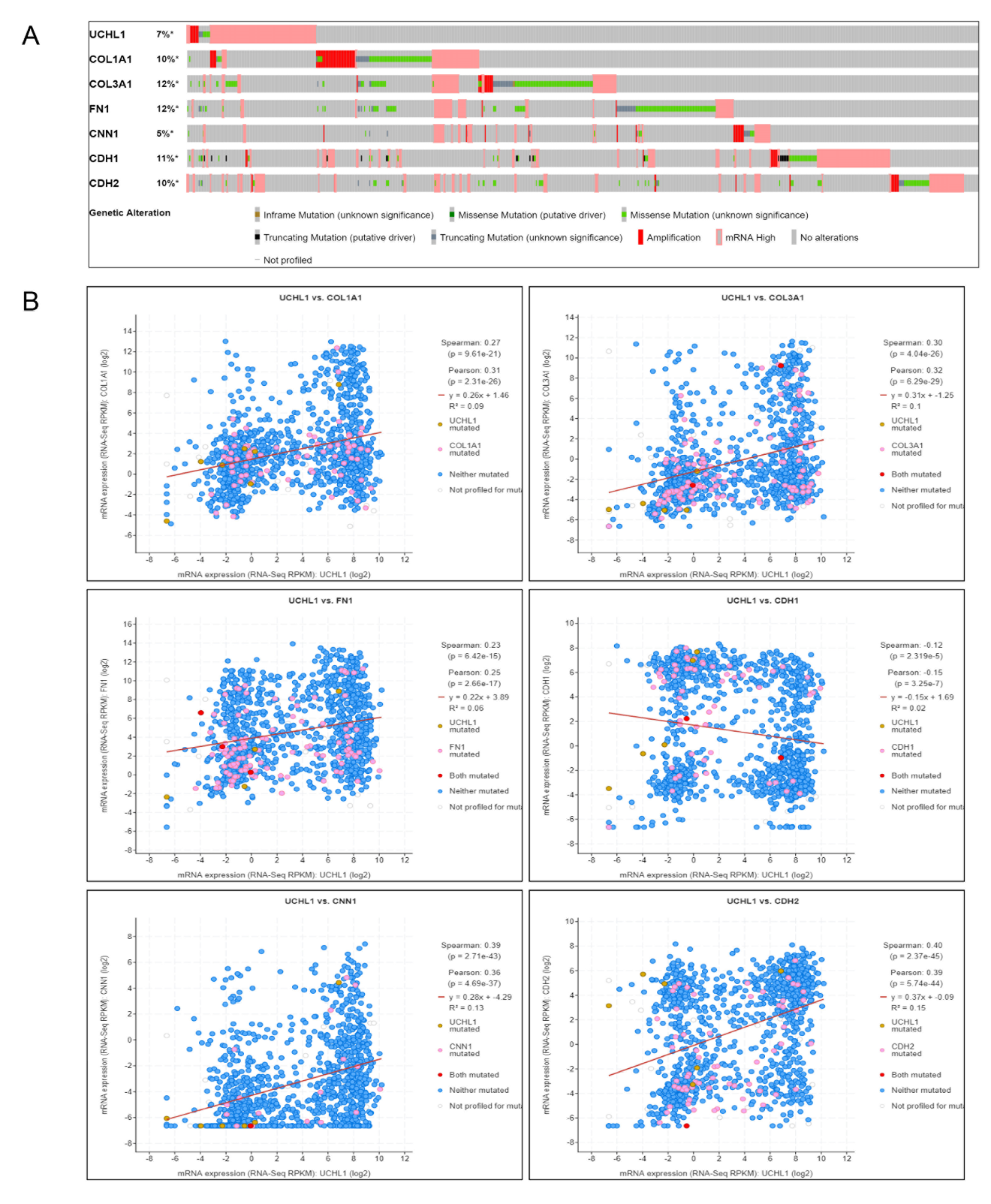


**Supplemental Figure 3. Co-expression of UCHL1 with marker genes of extracellular matrix.** (A, B) Histograms showing the co-expression (A) and levels (B) of UCHL1 and COL1A1, COL3A1, FN1 (fibronection), CNN1, CDH1, or CDH2 mRNA transcripts in Cancer Cell Line Encyclopedia, sourced from the TCGA provisional dataset (Broad, 2019) hosted at cBioPortal (cbioportal.org/datasets.jsp). This dataset was analyzed by RNA sequencing (included 1156 samples) and indicated here as RNA-seq RPKM (Reads Per Kilobase Million). Spearson and Pearson correlation as well as *p* value are noted on each graph.


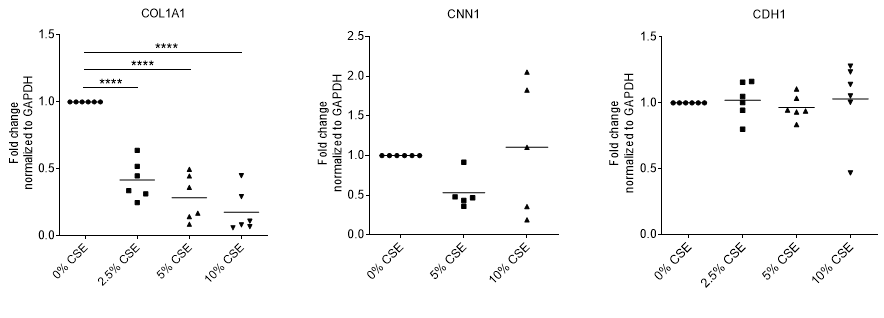


**Supplemental Figure 4. Expression of COL1A1 upon CSE exposure in PBECs from healthy lung transplant donors.** Cells were seeded to fibronectin/collagen pre-coated 12-well plates and treated with 0-10% CSE when the cells were 80% confluent. Cells were harvested for RNA isolation post 24 h (n=6). Data from different dose of CSE were normalized to 0% CSE. Significance was analyzed by One-way ANOVA, **** P< 0.0001.





**Supplemental Figure 5. Optimization of the approach to induce endogenous upregulation of UCHL1 expression.** (A, B). Induction of UCHL1 expression. BEAS-2B (A) and 16HBE (B) cells were transiently co-transfected with a total of 0.6 µg of the indicated sgRNAs and 0.6 µg dCas9-NED (dCas9 with No Effector Domain) or dCas9-VP64 (a tetramer of the viral VP16 transcriptional activator). Cells were harvested for RNA isolation at day 2 post transfection. Fold change of UCHL1 mRNA expression was normalized to dCas9-NED co-expressed with the respective sgRNAs (only shown for sgRNA (#1/2/3/4/5)). (C). Induction of UCHL1 expression in stable cells. 1 µg of plasmids expressing sgRNA (#4/5/6) or the negative control vector (pMLM.2.0) were transfected into BEAS-2B cells, stably expressing dCas9-VP64. mRNA expression of UCHL1 was measured at day 2 post transfection. (D) The expression of dCas9 after transient transfection of dCas9-VP64 or in stably expressing dCas9-VP64 BEAS-2B cells. (E) MS2 system in stable BEAS-2B cells. BEAS-2B cells stably expressing dCas9-NED or dCas9-VP64 were transfected to express sgRNA (#4/5/6) and MS2-NED or MS2-p65-HSF (in a 1:1 ratio, each 0.6 µg) (left panel). The right panel demonstrates the effect of a 2:1 ratio of MS2-p65-HSF and sgRNA (#4/5/6) (co-transfection of 0.8 µg MS2-p65-HSF and 0.4 µg sgRNA (#4/5/6) and others were transfected with a 1:1 ratio as indicated. At 48h post-transfection, cells were treated with or without 500 µg /ml hygromycin for another 4 days to select for positively transfected cells. (F, G). Selection based on fluorescence. BEAS-2B (F) or H1299 (G) cells were transiently co-transfected with 1 µg sgRNA (#4/5/6), 1 µg dCas9-mCherry-NED or GFP plasmid, and 1 µg dCas9-NED or dCas9-VP64. For mCherry/GFP-based enrichment, cells were collected 60 h post transfection and dCas9-mCherry-NED or GFP expressing cells were sorted by FACS. (H) BEAS-2B cells were co-transfected with 1 μg a mixture of UCHL1 sgRNA (#4/5/6), 1 μg dCas9-PRDM9-mCherry and 1 μg dCas9-Dot1l-mCherry. dCas9-NED-mCherry was monitored as a control. For mCherry-based enrichment, cells were harvested and sorted by FACS 48 h post transfection. The mRNA expression of UCHL1 was measured by real-time qRT-PCR (n=2). Statistical significance was determined using One-way ANOVA.


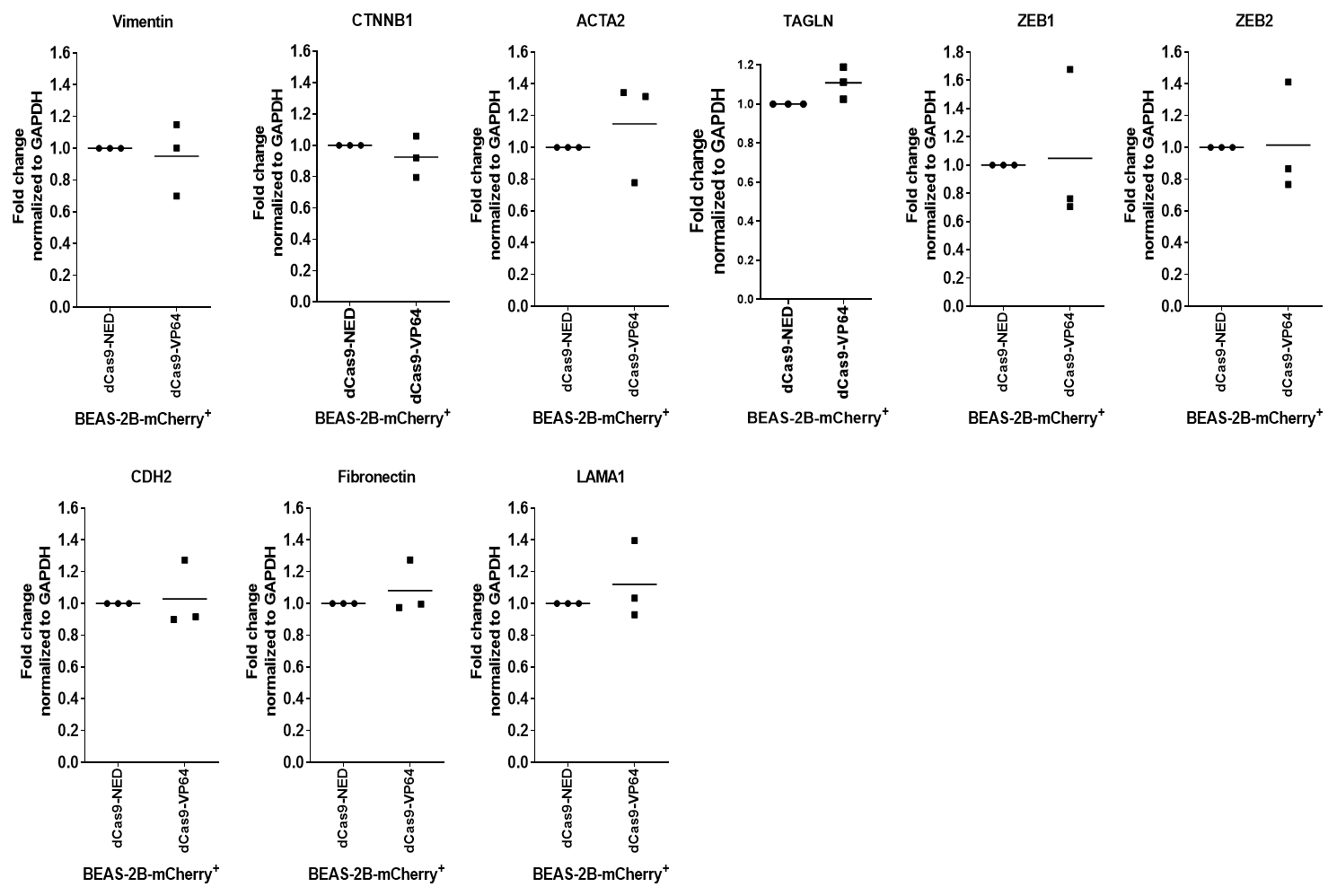


**Supplemental Figure 6. The expression of EMT markers upon targeted upregulation of UCHL1 expression.** BEAS-2B cells were seeded into 6-well plates, then transiently co-transfected with 1 μg a mixture of UCHL1 sgRNA (#4/5/6) together with 1 μg dCas9-NED-mCherry, and 1 μg dCas9-NED or dCas9-VP64 through PEI; For mCherry-based enrichment, cells were collected 48 h post treatment and mCherry-positively expressing cells were sorted by FACS. The mRNA expression of EMT-related genes was analyzed by real-time qRT-PCR (n=3). Statistical significance was determined using two-tailed unpaired *t*-test, **p*<0.05, ***p*<0.01, ****p*<0.001.

**
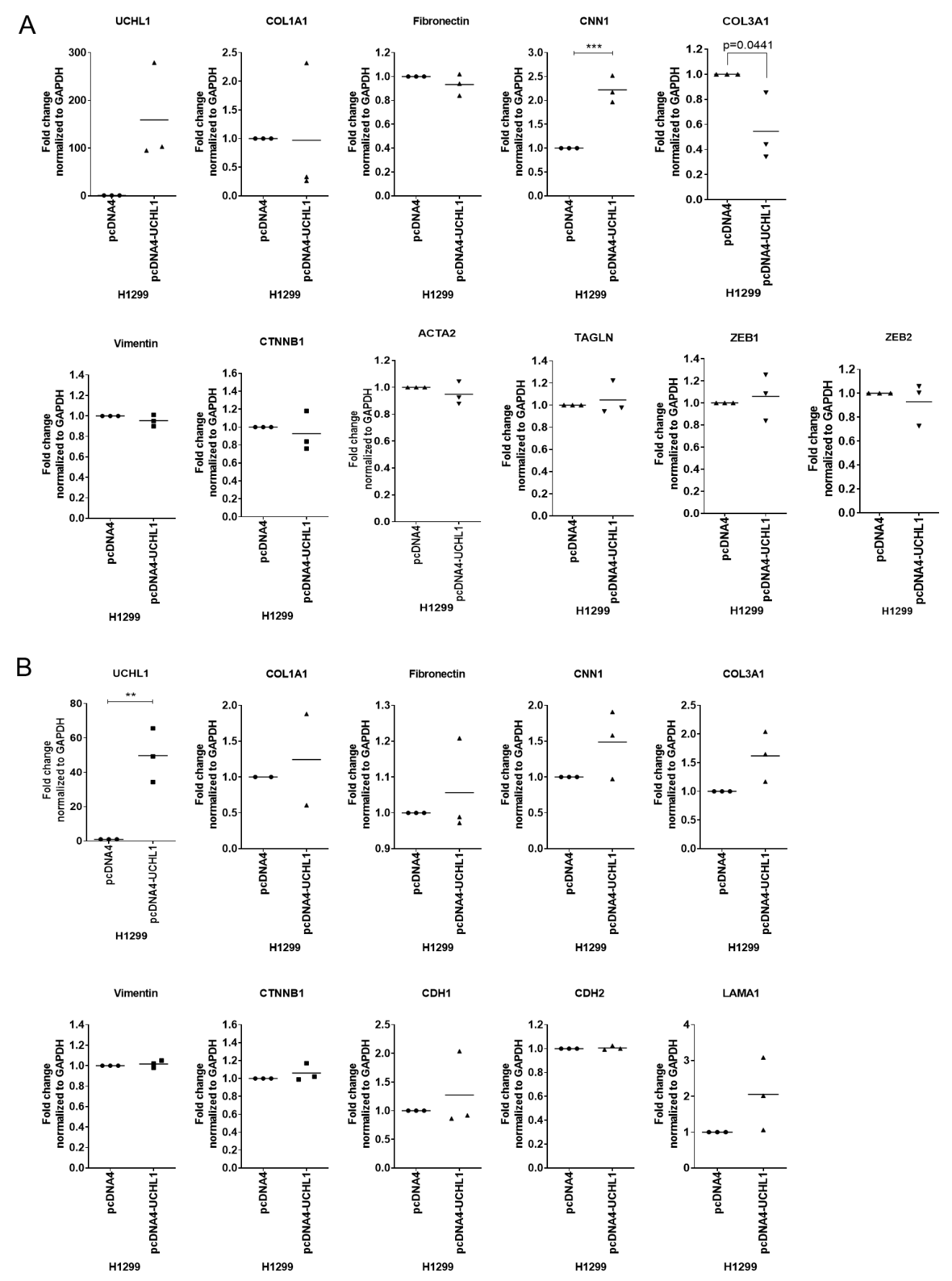
**

**
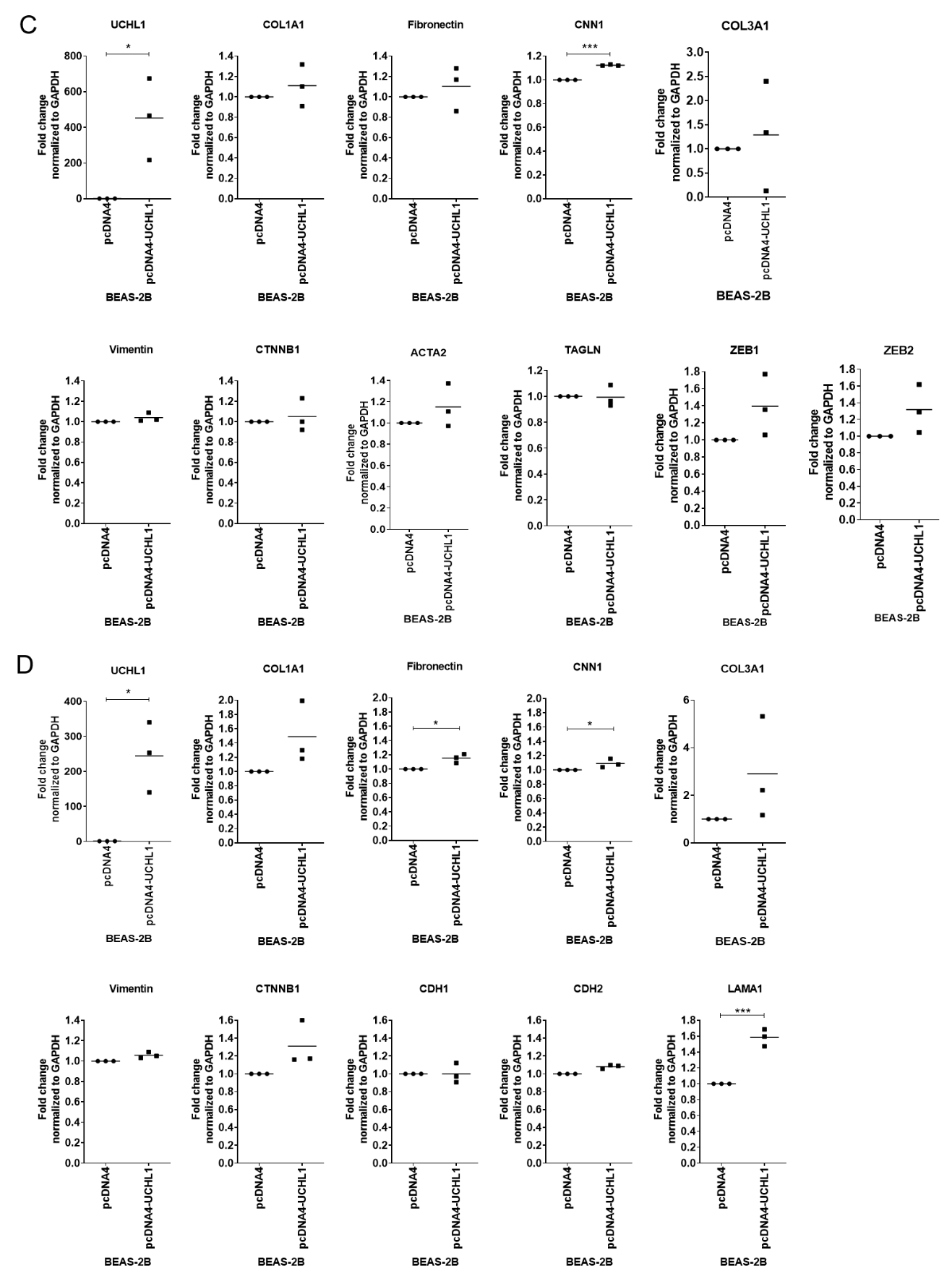
**

**Supplemental Figure 7. Exogenous upregulation of UCHL1 expression has no effect on the expression of EMT-related genes.** (A, B) H1299 cells were seeded to 6-well plates (5×10^5^ cells per well), then transfected with 3 μg pcDNA4 or pcDNA4-UCHL1 with 9 μl PEI when the cells were 80% confluence. Cells were harvested for RNA isolation at 24 h (A) or 48 h (B) after transfection. (C, D) BEAS-2B cells were transfected with 1.5 μg pcDNA4 or pcDNA4-UCHL1 with 4.5 μl PEI. Cells were harvested for RNA isolation at 24 h (C) or 48 h (D) post transfection. The mRNA expression of UCHL1, COL1A1, fibronectin, and EMT-related genes were analysed by real-time qRT-PCR. Three independent repeats are indicated. Statistical significance was determined using two-tailed unpaired t-test, **p*<0.05, ***p*<0.01, ****p*<0.001.


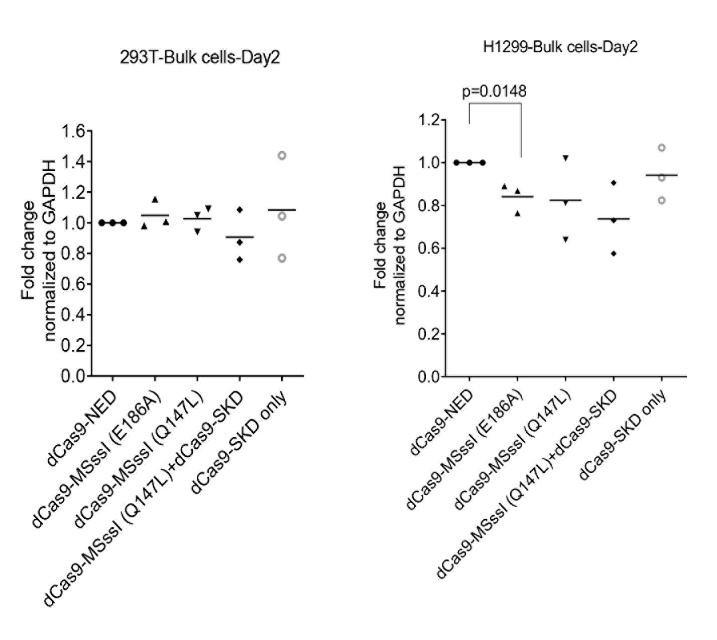


**Supplemental Figure 8.** **DNA methyltransferase tools to downregulate UCHL1.** HEK293T and H1299 cells were cotransfected with 1 µg UCHL1 sgRNA (#1/2/3) and 2 µg (in total) dCas9-ED (MSssI variants). Cells were collected 60 h after transfection, and the mRNA expression of UCHL1 was determined by real-time qRT-PCR (n=3). Statistical significance was determined using One-way ANOVA.

.



**Supplemental Figure 9. Targeted DNA methylation by dCas9-MsssI at TSS.** (A-B). H1299 (A) and HEK293T (B) cells were cotransfected with 1 µg UCHL1 sgRNA (#6/9/11/14) and 2 µg (in total) dCas9-mCherry-ED (MSssI variants, 1 µg each). Cells were collected 60 h after transfection and dCas9-mCherry-ED expressing cells were sorted by FACS for mCherry-based enrichment. Half of the sorted cells were harvested for short-term (day 2) expression measurements and the remaining cells were seeded in 24-well plates for long-term expression (day 12). Statistical significance was determined using two tailed t-test.


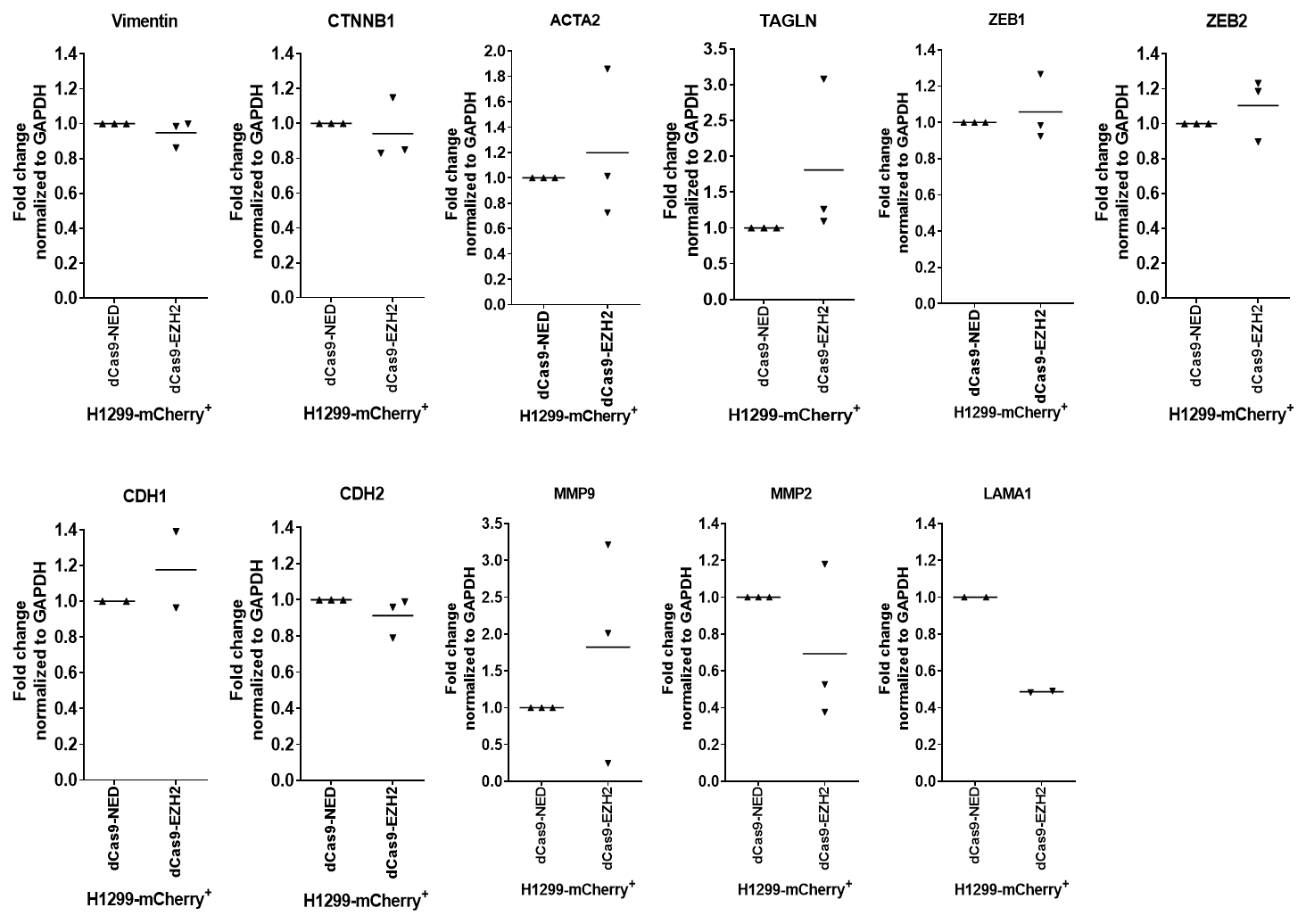


**Supplemental Figure 10. The expression of EMT markers upon the downregulation of UCHL1 expression.** H1299 cells were co-transfected with 1 μg a mixture of UCHL1 sgRNA (#1/2/3) and 2 μg dCas9-EZH2-mCherry or dCas9-NED-mcherry by PEI. For mCherry-based enrichment, cells were collected 48 h post treatment and mCherry-positively expressing cells were sorted by FACS. The mRNA expression of EMT-related genes was analyzed by real-time qRT-PCR. Three independent repeats are indicated unless stated otherwise. Statistical significance was determined using two-tailed unpaired *t*-test.


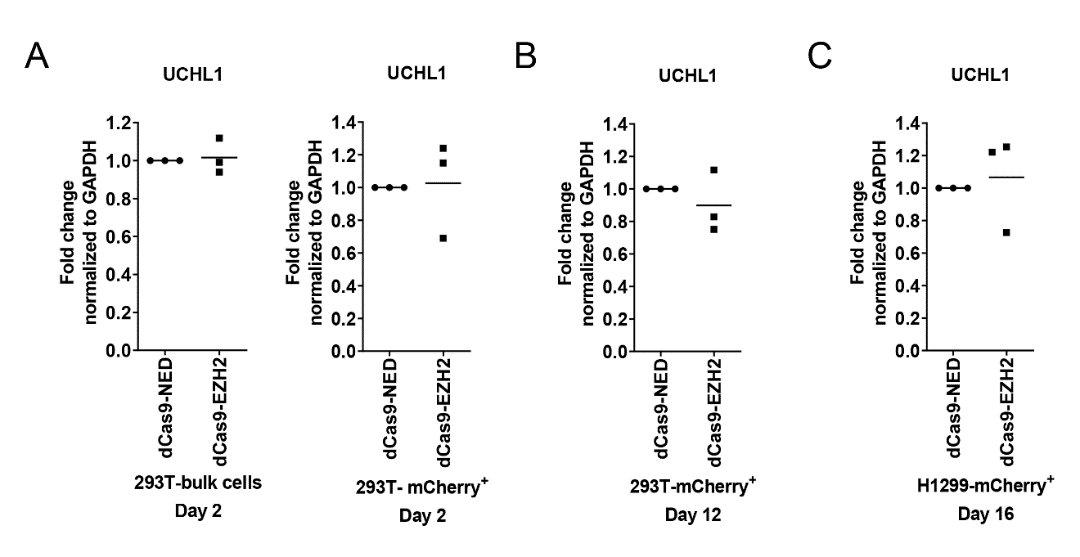


**Supplemental Figure 11. Targeted downregulation of UCHL1 by epigenetic editing EZH2.** (A-C) HEK293T or H1299 cells were co-transfected with UCHL1 sgRNA (#1/2/3) and dCas9-EZH2-mCherry or dCas9 -NED-mCherry by PEI. For mCherry-based enrichment, cells were harvested and dCas9-EZH2-mCherry expressing cells were sorted by FACS at 60 h post transfection. Bulk cells were collected before sorting. Half of the sorted cells were collected for short-term (Day 2) assessment and remaining cells were seeded into 24-well plates for long-term measurement (Day 12 for 293T cells and day 16 for H1299 cells). Total RNA was isolated with TRIzol and real-time qRT-PCR was performed to measure the expression of UCHL1.

**Supplemental Table 1. Correlation of UCHL1 mRNA expression and DNA methylation percentage of each analyzed CpG sites in different lung cell lines as indicated in Supplemental Figure 2A and 2B.** Correlation was established by nonparametric Spearman correlation test.

**
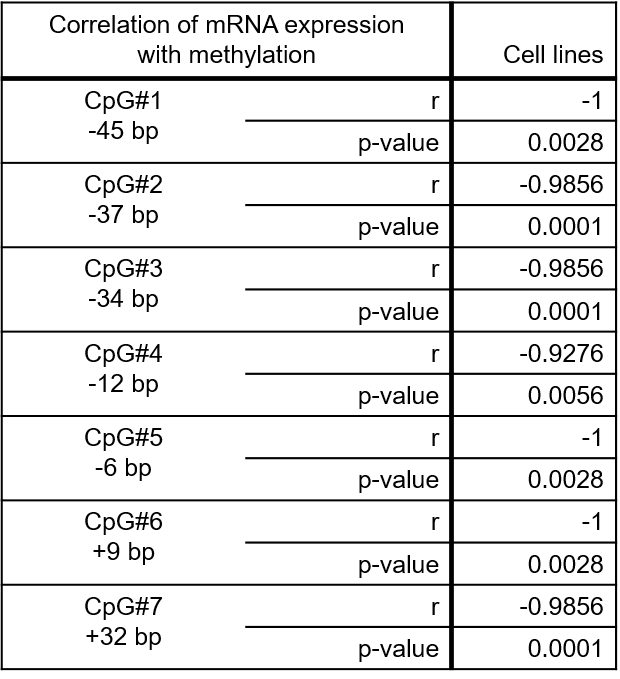
**

**Supplemental Table 2. List of sgRNA sequence targeting UCHL1**

|  | Target sequence |
| --- | --- |
| UCHLA sgRNA#1 | CTCTTGGAGCCCAGTTTAGC |
| UCHLA sgRNA#2 | GTCTCTTCACGGCTCCTCTA |
| UCHLA sgRNA#3 | CAGAAATACTTAGGTAGCGA |
| UCHLA sgRNA#4 | ACTCCCGCGGAAGAAATGGT |
| UCHLA sgRNA#5 | GTTAAGTCAGACCAGTACCG |
| UCHLA sgRNA#6 | CACTCGCCGGTGAGATAATC |
| UCHLA sgRNA#7 | TACCACTCCGCGCTGCGCAC |
| UCHLA sgRNA#8 | GCGCCCGGCAGAAATAGCCT |
| UCHLA sgRNA#9 | AACCGAACCGATCAGCGACT |
| UCHLA sgRNA#10 | GTGGCGTCTCGCGCCGTCTC |
| UCHLA sgRNA#11 | GACTCGAGCTTTAGAGTAAT |
| UCHLA sgRNA#12 | ACGGGGGGTTCGTACCCATC |
| UCHLA sgRNA#13 | AACAGCTAGCGGAGCCGCCC |
| UCHLA sgRNA#14 | ACTCTACGAAACCGGTCACG |
| UCHLA sgRNA#15 | ACTCGGCTGCACGGGCTTCG |
| UCHLA sgRNA#16 | GCTGTGTCATTGCGCCGGCC |
| UCHLA sgRNA#17 | CGCCGGCCAGTGGCGCTTCG |
| UCHLA sgRNA#18 | TTAACTGAAGCACCGTCCTA |
| UCHLA sgRNA#19 | GAGCAACCATGATGACTCGG |
| UCHLA sgRNA#20 | ATCAGATTTATCGAGCGCCT |

**Supplemental Table 3: Information of PCR primers used in the supplement**

| Primer | Sequence (5’-3’) | Application |
| --- | --- | --- |
| Vimentin-Fw | TACAGGAAGCTGCTGGAAGG | qRT-PCR primer for Vimentin |
| Vimentin-Rv | ACCAGAGGGAGTGAATCCAG |  |
| CTNNB1-Fw | GTCCGCATGGAAGAAATAGT | qRT-PCR primer for CTNNB1 |
| CTNNB1-Rv | GCACAAACAATGGAATGGTA |  |
| ACTA2-Fw | CTGTTCCAGCCATCCTTCAT | qRT-PCR primer for ACTA2 |
| ACTA2-Rv | TCATGATGCTGTTGTAGGTGGT |  |
| TAGLN-Fw | CTGAGGACTATGGGGTCATC | qRT-PCR primer for TAGLN |
| TAGLN-Rv | TAGTGCCCATCATTCTTGGT |  |
| ZEB1-Fw | GCCAACAGACCAGACAGTGTT | qRT-PCR primer for ZEB1 |
| ZEB1-Rv | TCTTGCCCTTCCTTTCCTG |  |
| ZEB2-Fw | AAGCCAGGGACAGATCAGC | qRT-PCR primer for ZEB2 |
| ZEB2-Rv | CCACACTCTGTGCATTTGAACT |  |
| CDH2-Fw | GGCTTCTGGTGAAATCGCAT | qRT-PCR primer for CDH2 |
| CDH2-Rv | AAGAGGCTGTCCTTCATGCAC |  |
| LAMA1-Fw | ATGGAAAATGGCACACTCTT | qRT-PCR primer for LAMA1 |
| LAMA1-Rv | AGACTGGGTGTGTGGACTTT |  |
| MMP2-Fw | GTTCCCCTTCTTGTTCAATG | qRT-PCR primer for MMP2 |
| MMP2-Rv | CTTGCCATCCTTCTCAAAGT |  |
| MMP9-Fw | GACGATGACGAGTTGTGGT | qRT-PCR primer for MMP9 |
| MMP9-Rv | GAAGATGAAGGGGAAGTGG |  |
